# What Is Next in This “Age” of Heme-Driven Pathology and Protection by Hemopexin? An Update and Links with Iron †

**DOI:** 10.3390/ph12040144

**Published:** 2019-09-24

**Authors:** Luis Montecinos, Jeffrey D. Eskew, Ann Smith

**Affiliations:** 1 Department of Physiology & Biophysics, School of Medicine, University of Chile, Santiago 838-0453, Chile; lmontecinos@med.uchile.cl; 2 KITE Pharma, Inc., Santa Monica, CA 90404, USA; Jeskew@kitepharma.com; 3 Department of Biochemistry & Molecular Biology, School of Biological & Chemical Sciences, University of Missouri-Kansas City, MO 64110, USA

**Keywords:** hemopexin, heme homeostasis, iron homeostasis, hemolysis, haptoglobin, ferroptosis, inflammation, biomarker, heme oxygenase, liver, microbiome, trauma, hemorrhage

## Abstract

This review provides a synopsis of the published literature over the past two years on the heme-binding protein hemopexin (HPX), with some background information on the biochemistry of the HPX system. One focus is on the mechanisms of heme-driven pathology in the context of heme and iron homeostasis in human health and disease. The heme-binding protein hemopexin is a multi-functional protectant against hemoglobin (Hb)-derived heme toxicity as well as mitigating heme-mediated effects on immune cells, endothelial cells, and stem cells that collectively contribute to driving inflammation, perturbing vascular hemostasis and blood–brain barrier function. Heme toxicity, which may lead to iron toxicity, is recognized increasingly in a wide range of conditions involving hemolysis and immune system activation and, in this review, we highlight some newly identified actions of heme and hemopexin especially in situations where normal processes fail to maintain heme and iron homeostasis. Finally, we present preliminary data showing that the cytokine IL-6 cross talks with activation of the c-Jun N-terminal kinase pathway in response to heme-hemopexin in models of hepatocytes. This indicates another level of complexity in the cell responses to elevated heme via the HPX system when the immune system is activated and/or in the presence of inflammation.

## 1. Introduction

The first direct evidence that hemopexin (HPX)-mediated heme transport links heme (iron-protoporphyrin IX) and iron metabolism was published in 1988 [1]. In that study, heme-HPX complexes were as effective as diferric-transferrin as a sole source of iron for the growth of mouse hepatoma cells that were used as models of liver parenchymal cells (i.e., hepatocytes). The rate of heme uptake via HPX is linked with cell growth and shows highest uptake in the period just before exponential growth and slowest in the stationary phase. Also, the iron status of hepatoma cells affects the extent of heme uptake. For example, decreasing intracellular iron with the chelator deferoxamine increased HPX-mediated heme transport, as did inhibiting iron-dependent ribonucleotide reductase (required for DNA synthesis).

Plasma HPX rapidly targets heme to hepatocytes [2] that respond with a cytoprotective program involving safe trafficking of heme, its iron, and, uniquely, also copper [3]. This copper is needed for the coordinate induction of heme oxygenase-1 (*HO1*) and metallothionein genes for antioxidant protection [4,5,6,7]. Within cells, heme delivered by heme-HPX endocytosis is quickly transported to the nucleus relieving Bach 1 repression, thus activating *HO-1* gene transcription [8,9]. Heme also travels to the smooth endoplasmic reticulum for degradation by heme oxygenases and into mitochondria, presumably via the mitochondrial heme exporter, FLVCR1b [10]. The heme-iron is utilized for the Fe/iron regulatory protein (IRP)/iron response element (IRE) system to regulate proteins of iron homeostasis at the translational level in part via storage on ferritin, thus keeping intracellular iron levels low [6]. Overall, the regulated delivery of heme by HPX maintains cell redox homeostasis. This is not the case when cultured cells are incubated with free “heme”, which is rapidly and extensively taken up compared with heme-HPX endocytosis (at approximately a five-fold higher rate on a molar basis) [11]. Heme generates ROS and, thus, is readily toxic [12]. Intracellular heme levels of 3 µM are rapidly reached, equivalent to 1 million heme molecules in the volume of the mitochondria [13].

The liver is the principle organ that responds to changes in systemic iron signals in order to maintain body iron homeostasis. Iron stores are regulated solely at the level of absorption not excretion. Interestingly, the bioavailability of heme from the diet as an iron source is superior to that of inorganic iron. However, iron, not heme, is exported into the systemic circulation because after [^59^Fe]heme is placed in the lumen of isolated rat duodenum, iron-transferrin not heme-HPX is present in the mesenteric vein [13]. This supports extensive catabolism of heme by HOs in duodenal enterocytes.

In addition, the liver is the first site of defense against dietary antigens and pathogens from the gut. Furthermore, low levels of heme are normally present in bile providing a source of heme and iron for gut bacteria and, thus, biliary heme is poised to influence the composition of the microbiome. In fact, using intravenous hemin to mimic a heme overload in the plasma of mice leads to the secretion of “black” bile [14].

The liver is an immune-responsive organ. Although, hepatocytes comprise ~90% of the liver mass, it also has many different types of cells including immunologically active Kupffer cells, stellate cells, and trafficking monocytes. As reviewed by Crispe [15], hepatocytes act in both innate and adaptive immunity. Hepatocytes synthesize and secrete several proteins needed for cell defenses during distinct pathologies ranging from ischemia, physical trauma, infections, and sepsis. These cells act not only by providing acute phase proteins in response to the cytokine IL-6 but also to direct T- cells [15]. They also respond to factors such as hepatocyte growth factor to synthesize IL-6. Rapid, short-term elevations in IL-6 are part of the early warning signals to activate the immune system in response to infections and injury. However, when IL-6 levels are sustained chronic inflammation occurs that can become life threatening, as in sepsis. Additionally, IL-6 is often dramatically elevated in patients receiving chimeric antigen receptor T cell therapy (CAR-T) and has been associated with both cytokine release syndrome (CRS) and neurotoxicity in these patients. Importantly, increased CRS grade was associated with peak IL-6 levels, peak ferritin (Ftn), and peak C-reactive protein [16]. Predictive models of CRS based on various cytokines and biomarkers are currently being researched [17].

Here, we provide preliminary data showing that IL-6 alters the response of models of liver cells to heme-HPX significantly limiting the extent of activation of the mitogen-activated protein kinase C-Jun kinase activation (JNK) pathway and changing levels of its substrates that are transcription factors. While the gene targets have not yet been identified completely, our data raise the possibility that when IL-6 is present in the short term, such cross talk may reprogram cell responses to heme-HPX that are normally beneficial. On the other hand, if certain protective signaling cell responses set in motion by the HPX system are attenuated by sustained IL-6, then there may be dire consequences. Such interactions between IL-6 and HPX may contribute to the worsening of sepsis including the development of septic shock. In the brain, these responses might exacerbate the pathology of intracerebral hemorrhage, traumatic brain injury or stroke where tissue and red blood cells injury is accompanied by inflammation.

The extent of damage by heme to the brain is beginning to be understood and several studies in mice provide evidence that HPX is protective in intracerebral hemorrhage (ICH), subarachnoid hemorrhage, and stroke. Hemopexin is present in human and rat CSF [18,19,20] and HPX mRNA has been located in ependymal cells [21], neurons [22], and glial cells [23]. Studies using HPX-null mice have revealed a role for HPX in myelin basic protein expression by oligodendrocyte and oligodendrocyte differentiation [23]. Hemolysis in the brain leads to disruption of the blood–brain barrier. However, a recent study [24] on patients with brain hemorrhage (intracerebral and subarachnoid) provides evidence that iron toxicity may be principally responsible for pathology rather than heme clearance. A review on heme in the context of neurodegeneration, where there are known mitochondrial defects, has recently been published in this *Pharmaceutical* special issue [25].

The purpose of this review was to assess from recently published research the biochemical role for HPX in protecting against heme toxicity in hemolysis and the accompanying inflammation in order to better define how heme- and iron-driven pathology develops in patients. Evidence supports that HPX is protective in both the presence and absence of bacterial/pathogen infection [26]. Hemopexin has two functions: firstly, as an extracellular antioxidant by sequestering heme and thereby protecting molecules in biological fluids from oxidation by heme and secondly by regulating heme uptake via heme-endocytosis which ensures redox metal homeostasis after heme and iron metabolism without leading to oxidative stress. Our focus was predominantly on recent clinical studies. Children are vulnerable to depletion of HPX because HPX is a developmentally regulated protein. Several studies included have investigated HPX as a candidate biomarker, generally in a panel with additional proteins; however, it is not always clear if the observed changes in HPX metabolism are linked solely to hemolysis or to inflammation or, as we contend here, to both. A more thorough understanding of the biochemistry of the HPX system may lead to novel therapeutic approaches for ameliorating or preventing heme-related pathologies. For example, the physiologically relevant role of the scavenger receptor LRP1/CD19 in the endocytosis of heme from Heme-HPX varies with cell type and is not fully understood in the context of the HPX system.

## 2. Mechanisms of Heme Toxicity

The cell damage by hemoglobin (Hb), by its heme (and by heme’s iron) are often ascribed to “oxidative stress”—an all-encompassing term for many different events encompassing inflammation and cytotoxicity. Both Hb and heme damage lipoproteins in the circulation [27,28]. Several recent studies show that high levels of heme consistent with that seen in patients with hemolytic disorders have several effects on cells. For example, heme inhibits the proteasome; generates aggresome-like induced structures (ALIS); activates the unfolded protein response (UPR); and activates several plasma systems including the coagulation cascade, binding to immunoglobulins and activating the alternative pathway of complement (see reviews by Roumenina et al. [29,30]).

Understanding these various aspects of heme toxicity for cells is of interest as well as assessing differences among various cell types (e.g., hepatocytes, macrophages, neurons, glia, endothelial cells, myocytes, kidney cells, and certain stem cells) in terms of their cell defenses. This information is also needed to better define panels of biomarkers in hemolysis, oxidative stress, and inflammation to aid in the diagnosis, prognosis, and response to therapy of patients with various hemolytic diseases and conditions.

### 2.1. Heme-Related Inhibition of the Proteasome

To identify the proteins and processes involved in heme stress, changes in the proteome phenotype of HO1^+/+^ and HO1^−/−^ mouse fibroblasts were investigated using mass spectrometry (MS) in combination with stable isotope labeling by amino acids of cultured fibroblasts in response to a range of heme concentrations [31]. The fibroblasts were incubated for 12 or 24 h with non-toxic levels of heme (5 or 10 µM) or with 40 µM that was deemed toxic and led to impaired mitochondria as evident by low ATP levels. Toxic heme caused nuclear condensation together with disorganization of the cytoskeleton. Furthermore, heme at levels greater than 10 µM induced caspase 3/7 activity, suggesting activation of apoptosis. It should be noted that 10 µM heme is often used in many in vitro studies in the literature. These heme-related toxicities were prevented by adding HPX to the cell culture medium. The MS analyses revealed 2068 proteins that responded to heme. Mass spectrometry MetaCore analyses showed protein networks involved in the UPR, general protein folding, and, as expected, anti-oxidant defenses. Also, heme stress, perhaps not surprisingly, had clearly made an impact on iron metabolism because the most strongly induced proteins were the proteins of iron storage H- and L-Ftn. Specifically, L-Ftn increased independent to initial HO1 protein levels in the two sets of fibroblasts. Heme had an impact on the proteasome because ubiquitin and the ubiquitin adapter protein sequestosome 1 (SQSTM1)/P62 were induced by heme. This is a scaffold protein with multiple domains central to a variety of functions. Principally, it is part of a signaling hub to control cell viability when there is cytotoxicity [32]. Various heat shock proteins and proteins involved in antioxidant defenses (e.g., peroxiredoxin, glutathione (GSH)-S transferase, and thioredoxin reductase) were also increased but to a lesser extent. As expected, heme levels were higher in the HO1^−/−^ cells but changes in response to toxic levels of heme were also found in cells expressing functional *HO1*, as well as in a murine macrophage cell line and human embryonic kidney cells. Intriguingly, heme bound the 19S ATPase subunit of the 26S complex of the proteasome. However, proteasome inhibition may not be due to the redox activity of heme because cobalt-protoporphyrin IX, which is redox inert, also inhibited the proteasome. The cell toxicity from heme was proposed to be due to the changes in proteasome structure likely from the effects of other reactive molecules such as lipid peroxides as well as from chemical reactions involving heme.

Consistent with these observations on the effects of high intracellular heme, it is not surprising that genetic hemolytic diseases are protein disaggregation disorders. In β-thalassemia, the erythrocyte precursors use protein quality control to protect themselves [33]. Due to the mutations in the *β-globin* gene in β-thalassemia, α-globin chains accumulate in erythrocyte precursors and precipitate due to the fact of their inherent instability. However, they are polyubiquitinated and degraded by the proteasome. Enhanced proteasome activity due in part to the induction of protein subunits of the proteasome is found in cells from β-thalassemic patients. This was shown by transcription profiling to be due to the stress response transcription factor Nrf1. Brief inhibition of the proteasome led to α-globin chain accumulation; however, α-globin did not accumulate in the erythrocyte precursors of β-thalassemic mice treated with the proteasome inhibitor bortezomib. Thus, the means to regulate protein quality control may differ between erythroid precursor cells in vitro and in vivo in mice perhaps due to the differences in activity of the Hb stabilizing protein.

### 2.2. Heme-Related Aggresome-Like Structure Formation

In 2016, Travassos and colleagues [34] were the first to report that heme, at high levels (30–100 µM), would produce protein aggregates intracellularly. These aggregates also known as aggresome-like induced structures (ALIS) had a distinct composition, which included ubiquitinated proteins and SQSTM1/P62 in RAW macrophages [34]. The transcription factor NF-E2-related factor (NRF2) was required, which increases the transcription of SQSTM1/P62 raising protein levels. By using the HO1 inhibitor tin-protoporphyrin and cells derived from mice lacking FtnH, these ALIS were shown to be formed in response to oxidative stress from the intracellular heme and its iron. Furthermore, the response to heme was reversible because there were changes in the number of the ALIS over time. Interestingly, the maximum formation occurred 12 h after heme and declined over the following 12 h. Importantly, this indicates that there is an active cell recovery response to clear these structures. Even at these high levels, heme did not activate autophagy—the cell survival mechanism that allows the controlled degradation and replacement of cell components and can induce cell cycle arrest and inhibit apoptosis.

### 2.3. Heme-Related Unfolded Protein Response (UPR)

During the course of atherosclerosis, plaques undergo defined changes as the pathology develops. Ultimately, this can lead to a high risk of acute ischemic events. Plaque instability is increased depending upon the lipid content and the presence of a thin fibrous cap. Intraplaque hemorrhage occurs after activation of neovascularization and new bloods vessels enter the plaques. When these plaques (i.e., atheromas) rupture, both smooth muscle cells and resident macrophages are exposed to high heme levels, to oxidized forms of cell-free Hb, and to the accompanying oxidative stress.

Addition of heme to cells in vitro increases intracellular reactive oxygen species (ROS) [35] and evidence supports that oxidatively damaged proteins are likely to be partially misfolded, thus generating endoplasmic reticulum (ER) stress. When this occurs, the UPR (for reviews, see References [36,37]) is activated to prevent ER overload, restore homeostasis in the ER, and to promote cell survival. The UPR consists of the activation of several intracellular signal transduction pathways and operates at both transcriptional and translational levels. For example, mobilization of the transcription factors ATF6, ATF6B, CREB3, and the inositol-requiring enzyme 1 leads to increased transcription of genes encoding chaperones such as HSP90, HSP70 and GRP94. Mobilization of the pancreatic ER kinase-like ER kinase (PERK, pancreatic eIF2α kinase, EIF2AK3) leads to phosphorylation of the translation initiation factor eIF2α, leading to a global downregulation of protein synthesis.

The analysis of human carotid artery specimens from patients undergoing carotid endarterectomy showed significant amounts of heme in atherosclerotic plaques Human aortic smooth muscle cells are key players in the development of atherogenesis. To evaluate how high levels of heme cause ER stress and activate the UPR, the levels of markers of ER stress were measured after human aortic smooth muscle cells were incubated with heme for up to 6 h [38]. In cultured cells isolated from complicated lesions with hemorrhage, even those obtained some distance from the border of intraplaque bleeding, had increased expression of glucose-regulated protein 78kDa (Grp78) and CCAAT-enhancer binding protein homologous protein (CHOP) in response to heme compared with atheromas or healthy arteries. The CHOP is a multi-functional transcription factor that down regulates the anti-apoptotic mitochondrial protein BCL2 which is an apoptosis regulator supporting pro-apoptotic activities of the mitochondria; and, thus, a robust marker for apoptosis. Low levels of heme (1 µM) did not activate the UPR; however, heme at 10 or 25 µM, activated all three “arms” or “sensors” of the UPR. Inositol-requiring enzyme activation, PERK activation, and ATF6 activation were detected after 3 h of incubation. These data support that, in complicated lesions from patients with atherosclerosis, heme triggers ER stress and cell death.

Based on these changes in UPR marker proteins in response to the high heme, a model was presented that heme, depending upon its concentration, activates both pro-apoptotic and pro-survival pathways. Significantly, incubating human smooth muscle cells in vitro with heme caused a transitory pro-apoptotic response and permanently activated the pro-survival responses (ATF6, GRP78). There was also a high level of induction of heme-responsive genes including *HO1*, which is cytoprotective. This research provides evidence for interaction between heme and iron metabolism together with the UPR activating pathways to help cells survive heme-induced stress. Furthermore, α-1 microglobulin and HPX, which both bind heme in plasma, significantly decreased the effects of heme on ER stress markers when added to the medium of human smooth muscle cells in vitro. Thus, patients with atherosclerosis may benefit from plasma replenishment with HPX.

### 2.4. Heme-Related Activation of Complement

The complement system consists of ~70 proteins and is another part of the human immune system that contributes to immune defenses but can also exacerbate inflammatory diseases and conditions. Complement proteins rapidly sense tissue changes and the antigens presented by invading pathogens. By binding to these molecular signals on the surface of pathogens, they facilitate pathogen destruction by bringing in phagocytic macrophages and neutrophils that also produce cytokines [39]. Complement signaling and activation pathways have now been linked with T cells, which act in homeostatic responses.

Merle and colleagues [29] showed that the complement system is activated in sickle cell disease (SCD) patients and is the likely cause of nephropathy because deposits of complement components C3 and C5b were present within the tissues of kidney biopsies taken from patients. Using a combination of in vitro and in vivo approaches with a mouse model of SCD, the mechanism of complement activation triggered by intravascular hemolysis was shown to be due to the effects of heme on endothelial cells. Furthermore, intravenous (IV) HPX was protective limiting the complement activation.

In a mice model of phenylhydrazine (PHZ)-induced intravascular hemolysis (IVH), C3 deposits were detected predominantly in the kidney and in the glomeruli. The role of C3 was confirmed because the renal injury was markedly less in C3 null mice. In WT mice, IV hemin or IV purified human Hb also led to activation of complement with extensive C3 deposits in the kidney. Significantly, there were no detectable deposits after HPX was given IV providing strong evidence that HPX effectively prevented complement activation in response to heme and, importantly, also to Hb. As an avid heme-binding protein, this protection by HPX also supports that the pathological effects of Hb are due in part to the release of heme with activation of complement. In contrast, human serum albumin that can bind two molecules of heme but significantly lower affinity than HPX, was not protective. Phenylhydrazine causes a huge IV Hb/heme load with extensive acute kidney injury as shown by tissue and vascular markers of inflammation (e.g., IL-6, P-selectin), as well as ultrastructural changes in the renal tubules. Surprisingly, this kidney damage was resolved and renal function restored without treatment. In contrast, these changes in the kidney did not occur in response to IV heme. In addition, HPX was not protective in the PHZ-treated mice that were dying possibly from acute pancreatitis [40]. While the overall conclusion was that HPX is a sentinel for the kidney in IVH against heme toxicity, it was suggested that events generating hemolysis-derived products before the release of heme might be the damaging species. Degradation of RBCs produced heme-loaded microvesicles and heme induced both the alternate and complement pathways and activated complement either in serum in vitro or on endothelial surfaces. Complement proteins can be inactivated with blocking antibodies, although this type of treatment is still under development. Nevertheless, the identification of a role for complement in vascular injury and organ pathology raises the possibility that such antibodies would be useful as adjunct therapeutics with plasma protein replenishment to minimize or preclude development of the pathology of heme toxicity.

### 2.5. Hb-Related Globin Toxicity

It has been known for some time that globin is potentially toxic to cells. Hb (from RBCs) and myoglobin (from myocytes) are the principal sources of globin in plasma. There are clear differences in the structure of these two molecules that drive their chemical responses to agents such as CO, which is released only from heme catabolism [41] and from lipid peroxidation. Due to the fact of its inherent instability, α-globin aggregates within red blood cells at various stages of their development and sites in the body causing much of the pathology in β-thalassemia (see Section 2.1). An excess of α-globin chains without sufficient β-globin due to the point mutations in this disease is potentially toxic and to counteract this, an α-globin chain stabilizing protein is expressed in erythroid precursor cells solely, which is a chaperone for the α-globin [42].

The mechanism by which heme itself and Hb contribute to cell and protein damage in hemolysis has been extensively investigated during the past ten years or so and is well understood. Early studies on the transfer of heme to HPX from Hb in vitro revealed denaturation of the globin, apparent in the absorbance spectra [28]. In vivo studies with mice or rats or in vitro studies with non-neuronal cells have not yet pinpointed globin as a toxic agent. However, a recent in vitro study with mixed mouse neurons and glia in culture incubated with hemoglobin (Hb) and HPX (i.e., in the absence of haptoglobin (Hp) led to white precipitates of globin protein in the medium on the surface of cells and within cells [43]. This was associated with significant levels of neurotoxicity, based on lactate dehydrogenase release from neurons, although not glia, after incubation with Hb (4 µM, i.e., 16 µM heme) and HPX (1mg/mL, i.e., ~17.5 µM). When the mixed cells were incubated with heme, HPX completely protected both types of cells from heme toxicity. Protective effects of HPX were not considered due to the extracellular “scavenging” but rather were ascribed to intracellular protection against heme toxicity via heme breakdown by HOs and protection against iron toxicity by Ftn, which was shown to be induced [44,45].

Published studies on Hp knockout mice have not recorded obvious globin precipitation, not even after PHZ-induced hemolysis, which is extensive, and not even after *IV* HPX. Also, Hb clearance from the plasma occurs at essentially the same rate in Hp-null mice and wild-type mice without obvious toxicity [46]. However, Hb clearance in mice differs from humans because although human Hp binds to CD163, mouse Hp does not and mouse Hb binds more tightly to CD163 than human Hb [46]. Without Hp, the kidney and muscle of mice [47] are the tissues most affected by oxidative damage in response to Hb. The globin toxicity detected in neuronal cells in vitro may be due to the very defined medium that may not contain the proteins and other molecules normally present in CSF (or at their normal concentrations). Perhaps further research on Hp-null mice and human brain cells will help determine if there are protective factors in cells and biological fluids in vivo, for example, other heme binding molecules that mitigate against globin precipitation or oxidation reactions, or if there are additional differences to be discovered between heme and Hb clearance in humans and mice.

### 2.6. Heme and Iron-Dependent Cell Death by Ferroptosis

There are clearly defined programs and mechanisms for cell death such as apoptosis or necroptosis in response to toxic agents and/or a variety of environmental conditions and stresses. Distinct morphological changes including abnormal mitochondrial size may also be manifest. An iron-dependent cell death pathway termed ferroptosis has been investigated and defined [48,49,50]. This requires intracellular oxidative stress that increases lipid peroxidation, decreases GSH levels, thus changing GSH/GSSG and increasing γ-glut amyl transferase, together with the instigator iron. Ferroptosis, as has been pointed out in Reference [49] has a distinct biochemical, morphological, and genetic “fingerprint”. Clearly, heme catabolism by HOs raises intracellular ferrous iron presumably predominantly in the cytosol. Several studies carried out with primary neuronal mouse cells, immortalized neuronal cells, and mice models of intracranial hemorrhage (ICH) [51] provide evidence that heme-mediated ferroptosis is part of heme toxicity in human hemolytic disorders. The responses of cells, including primary human hepatocytes [35], to heme-HPX where the intracellular redox state is maintained without ROS production have therefore been proposed to be anti-ferroptotic [3]. Also, studies with immortalized proximal renal tubule cells from HO1^+/+^ and HO1^−/−^ mice have provided strong evidence that it is not the generation of ferrous iron from heme catabolism solely that leads to ferroptosis. Furthermore, *HO1* can have an anti-ferroptotic role in these kidney cells [52].

Heart attacks and stroke occur after blood vessel injury that activates platelets causing occlusion and thrombosis. Also, platelet micro particles are seen in patients with hemolysis including SCD. Intriguingly, heme increased P-selectin expression that drives vaso-occlusion in SCD is evident by not only endothelial cells but also by platelets (see Section 4.1 and Section 4.2).

When human platelets suspended in Tyrode’s buffer were incubated with heme, filopodia-like structures appear at the cell surface in the presence of 5–10 µM heme [53]. Further changes in the cell surface consistent with damage—“blebbing”—were seen in response to 25 µM heme. Using sets of biochemical inhibitors and activators for various death pathways in conjunction with heme, the data supported that the platelets were neither undergoing apoptosis nor necrosis. There was a 5 fold increase in ROS and in markers of lipid peroxidation together with elevated HO1 and cytosolic iron in response to heme. In addition, oxidative stress was generated including effects on GSH with evidence for a role for the amino acid antiporter system Xc, which transports cystine into cells that is needed and used for GSH synthesis. The ROS production (i.e., increased hydroxynonenal) by heme was controlled when the iron-chelator deferoxamine, the ferroptosis inhibitor ferrostatin-1 or the HO inhibitor tin-PPIX were present. Thus, 10–25 µM heme generated ferroptosis in platelets in vitro.

Protection of neurons against ferroptosis may come from the development of inhibitors of double-stranded RNA-dependent protein kinase, as shown recently in mouse hippocampal H22 cells [54]. This kinase responds to oxidative stress and ER stress and may be part of the pathology of neurodegeneration with mitochondrial impairment as in Parkinson’s disease and Alzheimer’s disease that are both associated with increased brain iron [55,56,57].

Brain damage after ICH includes irreversible damage to neurons in primary and secondary injury, the latter is generally considered to be due to the Hb and heme from lysed RBCs. Apoptotic cells and necrotic cells have both been identified in the perihematomal region after ICH. Both ferroptosis and necrosis were identified as being involved in Hb- and heme-induced toxicity in primary cultured neurons, immortalized hippocampal neurons (HT22 cells), and in male mice after ICH [58].

Human monocytes may also be susceptible to ferroptosis induced by heme in transfusion-dependent patients, who often develop iron-overload. When human monocytic THP-1 cells in serum-free medium were incubated for 2 h with 20 µM heme, there was an increase in ROS production together with an increase in cells with markers of necrosis but not markers of apoptosis [59]. The ROS production was prevented by N-acetyl cysteine (which can chelate metals), by iron chelators deferoxamine and deferasirox (the latter being more effective), or an inhibitor of NADPH oxidase that generates ROS. Ferrostatin-1 decreased cell death in response to heme, whereas erastin, which induces ferroptosis by inhibiting the system Xc, increased cell death implicating ferroptosis as the cause. The protective effects of serum proteins against heme toxicity by binding heme was clearly shown because the addition of albumin or fetal bovine serum decreased the amount of heme associated with the cells and heme toxicity even at very high concentrations of heme (20–80 µM).

Overall, these studies demonstrate the potential that heme toxicity can kill cells by ferroptosis opening up an additional therapeutic approach in the clinic especially for neurodegenerative conditions and ischemic and hemorrhagic stroke. A review on the nexus between iron dyshomeostasis, excitotoxicity, and ferroptosis in stroke with a focus on ferroptosis has recently been published [60].

## 3. Potential Therapies to Replete HPX Levels for Protection against Heme Toxicity

As previously reviewed, HPX plasma levels can become depleted in patients with genetic hemolytic anemias (e.g., SCD, β-thalassemia) and hyper-hemolytic states for example after extensive blood transfusions [61,62]. In mice models of these hemolytic conditions, HPX replenishment by intraperitoneal (I.P.) or I.V. injection or by hepatic over-expression of HPX is often lifesaving. Thus, these animal studies formed the foundation for the concept of plasma protein replenishment therapies with HPX to combat the various damaging effects of heme in biological fluids. Unfortunately, Hp is often virtually undetectable in patients with hemolysis and so the current view is that a combination of HPX with Hp may prove most beneficial. Given the information on the effects of heme that include complement activation and activation of ferroptosis, additional combinations of therapies may prove even more beneficial to certain groups of patients.

### 3.1. Plasma Protein Replenishment Therapy

There are some unique aspects to the lipid profiles of patients with SCD, who overall have hypocholesterolemia, with low levels of both low- (LDL) and high-density (HDL) lipoproteins. In mice models of SCD, heme activates endothelial cells via toll-like receptor 4 driving events leading to vaso-occlusion [63]. Two independent studies of SCD patients describe differences in heme and HPX content of LDL and HDL [64,65]. Both addressed that oxidation of lipoproteins by heme and Hb is due to the ongoing hemolysis. In addition to HPX, α-1 microglobulin, albumin, and lipoproteins all bind heme. Vendrame and colleagues [64] considered that the varying levels of HPX would determine how much heme might intercalate in the lipoproteins in SCD patients. The LDL fraction had a higher heme concentration than did HDL, but the HDL fraction had a higher concentration of HPX. Thus, HDL is considered an important defense with potentially anti-inflammatory activity against heme toxicity but appears to be insufficient to protect endothelial cells from heme. Nevertheless, and intriguingly, HPX is linked with lipid metabolism because the cholesterol levels in the SCD patients were inversely correlated with circulating plasma HPX concentrations.

In the second study [65], the level of lipid peroxidation in SCD patients undergoing regular transfusions was measured using a sensitive assay—the end product of lipid peroxidation malondialdehyde. In SCD patients, Hp is essentially absent; however, HPX is present albeit significantly decreased. Consequently, as might be expected, both LDL and HDL oxidation were increased demonstrating the loss of protection against heme-mediated damage by the plasma proteins. At the postmortem of an SCD patient, oxidized LDL was found in the pulmonary artery. These observations reinforce the conclusion from other studies on SCD that replenishment therapy with HPX together with Hp may prove useful therapeutically; and this is also supported by studies with mice models of SCD (see Section 8).

### 3.2. Plasma Exchange

Plasmapheresis or therapeutic plasma exchange may provide another means to raise HPX and Hp plasma levels and may be more cost effective than recombinant proteins. Plasmapheresis is an established therapy and has been described as a symptomatic treatment for critically ill patients. Whole blood is removed, the plasma and cells separated, and the plasma replaced with another solution that may be specially prepared plasma from another person. Plasma is normally rapidly frozen after separation from RBCs and used after thawing within 24 h. The plasma is often used to remove and replace a patient’s plasma when they have autoimmune disease. Plasma exchange was effective in reversing organ dysfunction in children with thrombocytopenia-associated multiple organ failure [66]. However, while generally considered safe, it can cause bleeding, allergic reactions, and also potentially increase the chances of developing a bacterial infection. Using a small group of three patients, plasma exchange was found to successfully replete HPX and Hp in two SCD patients that were refractory to RBC exchange [67]. There were significant increases in both HPX and Hp while heme decreased ~30%. Unfortunately, the condition of one patient deteriorated, their circulating HPX and Hp levels were not increased by plasmapheresis presumably because their liver was not able to sustain and synthesize plasma proteins.

### 3.3. The Scavenger Receptor LRP1/CD91 as a Target for Therapies When There Is Hemolysis in the Brain

In a murine model of ICH, the administration of an antagonist (a potential inhibitor of agonist action) and agonists (producing a known effect) of TLR7 led to changes in mRNA and protein levels of the scavenger receptor LRP1/CD91 that binds heme-HPX tightly (Kd~4 nM, [68]). The expression of LRP1/CD91, HPX itself, and TLR7 were all increased when mice were treated after ICH induction with the TLR agonist imiquimod and decreased by TLR7 inhibitor ODN2088 [69]. The authors proposed that these studies reveal the potential for a role for TLR7 in the uptake of heme-HPX, although this was not shown directly. (See also Section 8.5.)

## 4. Protective Functions of HPX in Various Pathologies

The published clinical research on HPX within the past two years covers diverse clinical states: SCD, β-thalassemia, thrombolytic conditions, trauma with hemorrhage, intracerebral hemorrhage, hemorrhagic stroke, spinal cord injury, carotid artery disease, tuberculosis and diabetes, liver disease, kidney injury, pre-eclampsia, and microbial invasion of the amniotic cavity. In several studies, HPX has been investigated as a biomarker, used as a parameter to establish hemolysis or, in some cases, to assess the extent of hemolysis (“hemolytic index”). Depending upon the cause, extent, and duration of hemolysis there are significant differences in the amount of heme load from Hb that HPX counteracts. Additional factors arise when the immune system has been activated. Intriguingly, there are compensatory changes in the immune system in depression, bipolar disorder, chronic widespread pain and fatigue, and HPX expression in the CSF is increased in these patients. Notably, HPX is not an acute phase reactant in humans unlike Hp [70]. Furthermore, even when there is extensive or chronic hemolysis, as in SCD, high levels of Hb readily deplete plasma Hp but HPX is often still present albeit at lower than normal levels. Evidence from both clinical and animal research now supports that changes in HPX may differ depending upon the source of Hb, i.e., IV Hb or RBC lysis.

In patients with traumatic hemorrhage, high levels of heme and Hb (four times greater than heme) were present in plasma and heme levels were overall greater than 5 µM. Both were higher than plasma non-transferrin bound iron. Interestingly, the plasma heme levels were proportional to the number of units transfused rather than to the length of storage time of the RBCs. Thus, plasma transfusions are anticipated to provide significant HPX and Hp for protection against the Hb/heme from RBCs. Nevertheless, in the face of high levels of Hb, HPX levels rapidly and extensively decline. Information on HPX’s recovery time is needed in a variety of clinical situations and historical studies show that it may take several days [62]. In contrast to the recycling of HPX from liver, Hb–Hp complexes are degraded after endocytosis via the scavenger receptor, CD 163, in macrophages predominantly in the spleen. The extent of uptake by tissue resident macrophages including Kupffer cells in the liver is not known. When this Hb–Hp scavenger system is impaired or saturated, Hb–Hp remains in the plasma [71]. It is anticipated that in the clinic, information on the collective levels of heme, Hb, HPX, and Hp will prove important for assessing and determining patient treatments [72].

When using HPX as a biomarker, it is important to consider that HPX expression and its activity as a cytoprotectant differ with development, aging, and gender; as well as in response to the various diseases and conditions investigated. The age-dependent changes in HPX levels make newborns and young children vulnerable to heme-related pathology including oxidative stress and inflammatory responses, and can, under certain circumstances, drive inflammation. Furthermore, inflammatory responses generally are more prevalent with age and are exacerbated by genetic factors and by auto-immunity.

As described above (Section 2.4), the complement system is an integral part of the immune system and is composed of a plethora of plasma proteins that interact to fight infection in a variety of ways. Significantly, heme from Hb activates the complement system in vivo and in plasma in vitro. As with other immune system cells and proteins, this provides a means to protect in the early stages of disease including infections, and also to exacerbate inflammation and accompanying heme-driven pathology. These inflammatory conditions as well as the source of heme (IV heme, IV Hb or RBC lysis), determine which major organs and cells are affected. The lung is a more recently identified site of hemorrhage and heme injury, in addition to the liver, heart, kidney, and brain. Hemorrhagic brain damage weakens the integrity of the blood–brain barrier and heme is deleterious for the vascular system because of toxic effects on endothelial cells, smooth muscle cells, and, thus, blood vessel integrity.

### 4.1. HPX in Sickle Cell Disease

Unbiased analyses of the plasma proteome revealed several novel proteins that correlated with a therapeutic response (i.e., decreased hemolysis in young patients with SCD) during treatment with hydroxycarbamide (HC). Hydroxycarbamide is also known as hydroxyurea (an inhibitor of ribonucleotide reductase). As a long-time treatment for SCD, HC is thought to help prevent vaso-occlusion and vasculopathy by increasing γ-globin synthesis, altering adhesion factor expression by the endothelium, and by reducing the population of neutrophils. Plasma HPX levels significantly increased as the rate of hemolysis declined in response to HC, demonstrated by an increase in HbF% [73]. In addition, the levels of α-, β-, and γ-globin chains, the Hp-related protein, and complement C9 decreased. Thus, these protein changes were all associated with decreased hemolysis. Two other biomarker panels were investigated: one for inflammation (including ceruloplasmin, lower α1 acid glycoprotein, CD5 antigen-like protein, and factor XII among others), and another for decreased coagulation (including lower factor XII, carboxypeptidase, and platelet basic protein). Overall, the changes in these biomarkers also support significant improvements in these children in response to HC, which may prove useful therapeutically in other conditions with extensive hemolysis.

### 4.2. HPX Status in Platelets in Hemostasis and Thrombosis

When normal hemostasis is overwhelmed by pathological factors, there is uncontrolled clot formation that leads to blocked arteries or veins. Platelets together with endothelial cells and coagulation factors are crucial mediators of both vascular hemostasis and thrombosis. The thrombi that develop in arteries are rich in platelets and form at the sides of or around thrombotic plaques even where there is a high shear flow. Thrombi in veins have fibrin and RBC, but form on intact endothelial cell wall only in areas where there is a low shear force. Hemopexin may have specific roles in trauma injury with hemorrhage, which is one example of a thrombotic condition. A clot is also an example of thrombosis where there is a local obstruction of blood flow. Clots can also form on wounds and in those circumstances aid healing. Thus, depending upon the site of injury monitoring changes in HPX in plasma and other biological fluids may be a helpful in diagnosis, prognosis, and to predict the response to therapies in these patients.

In the setting of hemorrhage, hemostasis may occur by normal vasoconstriction and narrowing of blood vessels by an abnormal obstruction that includes atherosclerotic plaque via activation of the coagulation cascade (clot formation) or by physical ligation during surgery. These processes regulate vascular integrity and, thus, blood flow. The responses involve complex biochemical systems with multi-factorial processes that may even drive pathology in cancer. Hemopexin has been linked to various cancers (reviewed in Reference [74]) and approximately half of deaths of cancer patients with malignant tumors are associated with thrombotic events. Vascular occlusive diseases include atherosclerosis and lead to carotid artery disease and heart attacks, which is a leading cause of death in developed countries.

Evidence supports that the risk for thrombosis increases when there are larger platelets. Recent presumptive evidence supports that there are different populations of platelets, and two populations of human EDTA platelets were separated and identified based upon their mean volumes. Not only do platelets vary in size; but based on their diverse protein content that includes differences in HPX content, platelets have different functions [75]. Consistent with the link to thrombosis, large platelets were found to have more glycoproteins expressed on their cell surface and were found to be able to adhere better to collagen surfaces. Proteomic analyses revealed that 80/894 proteins differed in abundance between the large and small platelets. The differences among them are beginning to be defined. For example, the activation of integrin by ADP was greater in small platelets. Intriguingly, the proteins most relevant for heme and iron homeostasis including HPX, Hp and α-1 anti-trypsin, transferrin, and vitronectin, as well as immunoglobulins, were all more abundant in small platelets. It will therefore be interesting to understand the physiological relevance of these observations.

## 5. Hemopexin as a Biomarker for Concomitant Changes in Hemolysis, Inflammation, Heme, and Iron Metabolism—Some Challenges Include Age, Gender, and Type of Infection

Due to the developmental expression of HPX and of Hp and transferrin, the potential exists for key differences in the changes in these proteins as biomarkers (among other parameters of disease progression) between children and adults during therapy. This will be important especially in conditions complicated by inflammation in the presence of heme- (and iron-) toxicity known to be related to pathology. There will also be gender differences not solely because of estrogen-related effects in women but also to documented differences in iron-metabolism between men and women. Men with coronary artery disease have lower levels of plasma iron-transferrin and HPX but higher levels of inflammatory markers, macrophage infiltrates, and iron stores. There are increased levels of plasma Hb, increased numbers of Hb CD68^+/+^ macrophages, together with ferritin and transferrin receptor 1 in atheromas [76]. Thus, there are differences in both heme and iron metabolism in men and women with atherosclerosis. Such differences need to be fully established to help physicians better assess the progress of their patients from diagnosis through therapy.

Women are more prone to inflammatory diseases and are three times more likely than men to develop rheumatoid arthritis. Once more, this is not solely due to the presence of estrogen because several disease-associated genes reside on the X chromosome. Increases in inflammatory markers were recently found in the CSF of seven rheumatoid arthritis patients, which also correlated with fatigue. Fatigue is considered to be related to changes in the central nervous system. For over twenty years it has been known that tumor necrosis factor (TNF), which is an inflammatory cytokine, plays a major role in arthritis [77] and that targeting TNF (for example with a monoclonal antibody such as infliximab) is effective and improved the symptoms of fatigue. An “arthritis proteome” identified that TNF blockade with infliximab decreased 35 proteins in the CSF. Hemopexin was one out of seven candidate proteins and changes in HPX are known to be associated with arthritis [78]. Contactin 1 and complement factor B, both known to increase with systemic inflammation, were also decreased. Both fibrinogen γ-chain and complement factor B decreased in response to the TNF inhibitor. The decrease in HPX in response to infliximab was considered to be due to the amelioration of both systemic and CNS inflammation because markers of inflammation were decreased in the CSF. Overall, these data support a relationship between arthritis symptoms and CNS inflammatory pathways as targets of the TNF inhibition. Although the infliximab is stated to not cross the blood–brain barrier, TNFα crosses via receptor transport. Thus, systemic TNFα can damage the endothelial cells of the barrier creating localized permeability and infliximab peptides were detected in the CSF. Intriguingly, the circumventricular organs were proposed as an alternative route into the brain. These are brain structures with extensive and permeable capillaries, which allow the passage of molecules both into and from the brain (e.g., certain hormones [79]), and which contain cells with TLR4, a target of LPS and heme [80].

Pregnant women are at risk for heme-related toxicity when they develop the potentially lethal condition of preeclampsia, typically in the third trimester. Preeclampsia is associated with life-threatening high blood pressure among other symptoms and occurs in about 3–7% of all pregnancies. The pathology is not well understood but is thought to originate from changes in the placenta. It has been associated with autoimmune disorders and blood vessel problems, and there are several other risk factors including a history of diabetes, high blood pressure or kidney disease. Several studies have provided evidence that changes in the level of the heme-binding protein alpha-1 microglobulin (A1M) may correlate with the severity of this condition. As this condition becomes more severe, A1M levels increase and may reflect increased endogenous oxidative stress [81]. Hemopexin levels also changed in women with preeclampsia and may be related to changes in cardiac function. Hemopexin was proposed to become depleted in the early stages of pregnancy and to rise in the third trimester when the high blood pressure symptoms of preeclampsia become apparent. However, confirmation of this will require further analyses to fully elucidate. Nevertheless, biomarkers for Hb sequestration and degradation pathways may prove informative in assessing the mother’s status in preeclampsia.

### HPX as a Biomarker for Sepsis

Evidence from both clinical and animal studies [35,61] shows that HPX has potential as a biomarker in sepsis, as reviewed previously [62]. A panel of biomarkers is urgently needed for sepsis and septic shock patients due to the prevalence and high incidence of mortality and health issues. Patients who recover from sepsis endure long-term effects that include both physical and mental problems.

Vimentin may prevent lymphocyte apoptosis and be anti-inflammatory and, thus, may be a useful new target in sepsis [82]. Vimentin is one of the main constituents of the intermediate filament proteins that maintain cell shape, in part, by stabilizing the interactions of the cytoskeleton and perhaps more importantly by helping cells resist damage including apoptosis. Both serum and lymphocyte levels of vimentin were significantly increased in these two groups of patients. Hemopexin was one of 56 proteins identified in plasma, finally ranking fourth in a protein network of 12 based upon interactions with eight other proteins in the network (defined as “betweenness centrality”). Thus, HPX has been linked in a co-expression protein network with vimentin in patients diagnosed with sepsis and septic shock [82].

Ekregbesis and colleagues [83] tested the hypothesis that inflammation- or infection-associated hemolysis contributes to sepsis-associated anemia and also leads to detectable HO1 in plasma. Hemopexin levels were used as one of several parameters of hemolysis. The prevalence and extent of anemia, the presence of inflammation and levels of HO1 were determined in a cohort of ICU patients within 12 h of admission. Most patients (~83%) were deemed anemic and hemolysis was evident because of heme in the plasma (median levels ~21 µM), low Hp, decreased HPX (median levels 9.6 mg/dL, normal levels ~77 mg/dL) together with elevated HO1. Hemolysis was accompanied by IL-10-associated inflammation and the presence of IL-6 and TNFα. In this study, HPX was neither associated with the anemia nor with morbidity. However, all of the cytokines that were increased, including IL-6 and IL-10, were weakly inversely correlated with HPX. The IL-6 levels correlated with IL-10 levels and HO1 induction was associated with IL-10 levels rather than the extent of hemolysis. In contrast to other studies on sepsis [35,84], the severity of sepsis was considered better indicated by the rise in plasma IL-10 and the HO1 levels rather than the low HPX levels on admission.

Iron is needed by pathogens for cell growth and to establish an infection. Circulating ferritin light chain (L-ferritin, FtL), needed for iron homeostasis, has recently been shown to play a protective role in the cecal ligation and puncture model in mice that produces, as the gut contents leak, polymicrobial sepsis. [85]. There are known gender differences in iron markers and, in the clinic, serum ferritin levels together with several other markers are used to assess iron stores. Ferritin light chain is a secreted form of ferritin and low in iron content, which predominates in the circulation and whose function has been enigmatic for decades. The increase in serum ferritin in response to infection indicates a role in the acute phase response and this study supports such a link. The role of myeloid cell specific ferritin, using ferritin H (FtH) deleted cells, was also addressed. The FtH deficiency dampened the inflammatory response in vivo and decreased the response in vitro of bone marrow derived macrophages to LPS. However, it did not alter phagocytosis or bacterial clearance. Hemopexin levels have been implicated as protective in a previous study with this model of sepsis [35] but were not apparently affected by the loss of FtH in the myeloid cells (perhaps due in part to the wide range of individual values among the mice compared with those of Hp). FerritinH and FtL chains were both increased. FerritinL is anti-inflammatory because when it was elevated there were lower levels of liver injury, decreased inflammatory cytokine levels and increased inducible nitric oxide synthase, which is protective in the recovery from post-ischemic inflammation.

As noted by Chaim Hershko [86] in a review entitled “Iron, Infection and Immune Function”, the term “nutritional immunity” was first used in 1973 to demonstrate the importance of preventing human pathogens from multiplying and dividing to establish an infection [87]. It is well known that both iron and heme (the latter presumably as an iron source), are necessary for the growth of pathogenic bacteria. The need for heme is apparent from the plethora of uptake systems that bacteria employ to ensure the capture of heme, many of which are redundant ready to bind: heme itself, Hb, heme-HPX or Hb-Hp [88]. Therefore, HPX by sequestering heme acts in nutritional immunity and to overcome this heme/iron limitation many human pathogens express receptors for heme-HPX uptake. HPX has recently been linked to host immunity and the anti-microbial action of IL-22 because, in mice, limiting infection depends upon HPX but not on Hp, in spite of the fact that the expression of both proteins in the liver is induced by IL-22. Mice were infected with either *Escherichia coli* that causes sepsis in humans or *Citrobacter rodentium* that normally resides in the gut of mice and can also cause a wide range of infections in humans. *Citrobacter rodentium* shares several features with *E. coli* in how it establishes infections. After WT and IL22^−/−^ mice were infected, C-reactive proteins, serum amyloid A1, α2-macroglobulin as well as HPX and Hp were increased in plasma. Hepatic levels of HPX and Hp mRNA rose after infection in WT mice and in the IL22^−/−^ mice, but to a significantly lesser extent; consequently, Hb accumulated in the plasma due to the ongoing hemolysis caused by the infection. The presence of HPX was sufficient to clear *C. rodentium* in WT mice but not in HPX^−/−^, Hp^−/−^HPX^−/−^ or Hp^−/−^ mice. Furthermore, HPX reduced the level of infection (judged by decreased numbers of circulating bacteria) in IL22^−/−^ mice regardless of whether it was from an intravenous or oral infection.

## 6. Organs That Endure the Worst Heme Toxicity Include the Lung, Liver, Kidney and Brain

### 6.1. Lung Disease/Injury

Lung injury comes from exposure to many different kinds of inhaled compounds and can lead to pulmonary hemorrhage. Blood gets into the alveolar spaces when there is disruption of the alveolar capillary membrane (diffuse alveolar hemorrhage) [89]. The heme levels in plasma of patients with chronic obstruction pulmonary disorder (COPD) was significantly increased compared with non-smokers [90]. As described in Section 2.3, high intracellular heme levels lead to oxidative damage of proteins, which in turn activates the UPR. Consistent with this, the levels of one key regulator of the UPR, namely, GRP78, were extensively increased in the lung of COPD patients.

Chemical weapons were first used in World War 1; furthermore, chemical weapons such as chlorine may have been used in Syria within the past two years, while mustard gas and nerve agents were used against residents in Northern Iraq in 1988. The toxic gases chlorine and bromine damage lungs. Hemolysis developed in mice exposed to bromine by inhalation with heme-mediated lung damage that was ameliorated by a single IV treatment with recombinant human HPX [91]. Abnormally high levels of plasma heme were detected in the mice up to 14 days post bromine exposure. This was associated with increased lung elastase activity, which may induce the UPR via the PERK/CHOP arm [90]. The protection by exogenous human HPX was due to the counteracting high heme levels in part by sequestering heme, thus relieving RBC lysis after bromine exposure but also by stimulating the level of endogenous HPX circulating in plasma of these mice. The mechanism of the induction of endogenous HPX is currently unknown and may be occurring, but unrecognized or detected, in other mice models of hemolysis and HPX replenishment. Incidentally, bromine is present in cigarette smoke and, thus, represents a risk factor for chronic smokers.

Both these studies on patients support that HPX plasma replenishment is of potential therapeutic value in different kinds of lung injury that produce lung fibrosis and emphysema; and, especially, in combination with drugs that relieve the UPR and ER stress in lung cells.

### 6.2. Liver Disease/Injury

Many different types of liver pathology ultimately lead to liver cancer including chronic liver disease scarring such as cirrhosis. These pathologies can be caused by hepatitis B or C infection, by alcoholism or by fat accumulation (i.e., fatty liver). Hepatocellular carcinoma (HCC) is the most common type of primary liver cancer and there is a greater prevalence of HCC in men. Hepatocellular carcinoma can be cured if the liver disease is identified early enough. Hepatocellular carcinoma can develop when there is cirrhosis and the only cure is a liver transplant. Thus, identifying biomarkers of liver disease and especially HCC is of crucial importance.

Potential biomarkers including those for liver disease may be discovered in the content of exosomes, which are very small endocytic membrane bound vesicle derived from all cells (as far as is known). Exosomes were first discovered in maturing mammalian reticulocytes [92]. Their content of proteins and miRNA provides a window on the cells that release them providing information not only on the cell of origin but also potentially that of the tissues and organs from which the cell is derived. Thus, exosomes are providing useful biomarkers consisting of proteins and peptides that are readily being identified and quantitated by proteomic profiling. For example, HPX levels together with properdin were decreased in exosomes of patients with HIV with active drug use compared with HIV or with HIV patients with alcoholism. Properdin is the only known positive regulator of complement that stabilizes the alternative pathway convertases C3bBb [93]. Thus, HPX and properdin are poised to be potential markers for co-morbidity in drug abusers who were HIV positive, [94] indicating both liver disease and complement activation.

Several changes in carbohydrate structure of glycoproteins have been linked to liver disease [74] and another means to monitor liver disease utilizes identification of aberrant core fucosylation of plasma glycoproteins as biomarkers. Such alterations are readily assessed from patient serum samples. Human HPX has one O-linked oligosaccharide chain at its N-terminal threonine and five additional N-linked chains [95]. Core fucosylation of glycoproteins is site specific and clearly identifies a protein. A multiplex LC-MS/MRM array was used for the serological assessment of liver disease and to determine the extent of fibrosis of the liver. Key and specific changes (both increases as well as decreases in fucosylation of several N-linked glycoproteins) were detected at the stage of fibrosis in a comparison of the data garnered from healthy control, fibrotic, and cirrhotic livers. While five proteins were identified that marked liver fibrosis including transferrin, ceruloplasmin, α-1 antitrypsin, and vitronectin, there were also additional changes found in cirrhosis including HPX. Of relevance here, the changes included aberrant fucosylation at N630 of transferrin, N187 of HPX, and both N138 and N762 of ceruloplasmin, and N354 of clusterin [96]. Thus, detectable changes in fucosylation levels of several key plasma proteins including HPX, either increase or decrease as the liver disease worsens.

Additional monitoring of plasma proteins via glycan screening, including HPX [97], may also provide evidence of changes in liver function, thus aiding in the early detection and progress of HCC. This technique involves protein extraction using lectin-affinity binding of plasma samples. Preliminary data using MALDI-MS or ESI analysis show that glycan screens may prove useful to detect changes disease-related changes in HPX, Hp, and kininogen as liver function deteriorates.

### 6.3. Kidney Disease/Injury

It is well established that acute kidney injury occurs in hemolysis and that the kidney is susceptible to damage by Hb and heme. Thus, this organ is particularly vulnerable when plasma Hp and, eventually, HPX are both decreased.

Severe malaria pathology is associated with inflammation, endothelial cell activation and hemolysis. Furthermore, acute kidney disease is a known complication of malaria, especially in infection by Plasmodium falciparum and contributes to a high rate of mortality. One potential biomarker of acute kidney injury is chitinase-3 like 1 protein (CH3L1 YKL-40, HCgp39). It has also been associated with burn injury and bacterial infection. In spite of its name, CH3L1 is not an enzyme and does not act on chitin, which is an N-acetyl glucosamine polysaccharide that is expressed by endothelial and immune cells. However, CH3L1 on epithelial cells may bind chitin binding proteins expressed by bacterial strains that are potentially pathogenic. Thus, CH3L1 has been proposed to enhance the adhesion and binding of pathogenic bacteria. A high ratio of heme to HPX in children with malaria indicated severe disease with adverse clinical outcomes, and 46% of the children had acute kidney injury [98]. Interestingly, children with the highest heme levels (and lowest HPX) had significantly lower levels of parasitemia and the heme to HPX ratio decreased with recovery demonstrating a useful parameter to assess the severity of malaria.

Following up on these studies, Conroy et al. [99] investigated in a clinical trial if inhaled NO was an effective adjunct therapy for severe malaria in children in Uganda and investigated changes in CH31L as a potential biomarker for kidney damage. The rationale and hypothesis that children with malaria might benefit from exposure to nitric oxide (NO) as an adjunct therapy was first proposed in 2011. This was based on the response of adults with malaria to nitric oxide as a therapy, where NO minimized endothelial cell activation, data from animal models of malaria, and the improvement of adults with severe malaria in response to the NO precursor arginine. Plasma biomarkers were measured for 4 days and, finally, at day 14. There was an increase in CH3L1 in pediatric severe malaria (0–18 years of age) consistent with acute kidney injury. Several panels of plasma biomarkers were investigated including those for endothelial activation (Ang-2, slCAM-1). Hemopexin was included in the panel for intravenous hemolysis together with lactate dehydrogenase (LDH) and heme. The levels of CH31L correlated with markers of inflammation (e.g., surface receptor triggering receptor-expressed myeloid cells, [100]).

Kidney transplants are carried out worldwide and generally have a high success rate, although they do have risks. Nevertheless, markers for rejection after kidney transplantation are needed. Some research supports that in certain cases due to low abundance in the circulation, proteins may be more readily detected in urine than in plasma. Hemopexin, together with tetraspanin-1, has the potential to be a novel urinary exosome marker in adult T-cell-mediated rejection (TCMR) in kidney transplant recipients detected by nanoscale liquid chromatography coupled to tandem mass spectrometry. Tetraspanin-1 is a member of a family of 4-pass transmembrane proteins implicated in cell adhesion, migration and proliferation, which interact with membrane proteins including integrins. Large multimolecular complexes organize cell–cell interactions and matrix–cell interactions and consequently activate signaling pathways. Tetraspanin-1 is overexpressed in several different cancerous cells and plays a role in carcinogenic progression including the migration and invasion by malignant cells [101]. Intriguingly, both HPX and tetraspanin-1 were significantly increased in the TCMR patients [102].

### 6.4. Brain/Injury and Neurotoxicity

The brain is protected by the integrity of the blood–brain barrier that prevents toxic plasma components like heme, intact blood cells, and pathogens from reaching the brain. However, most brain injuries including stroke and hemorrhage (intracerebral, subarachnoid, stroke) cause damage within the brain that leads to damage of the protective blood–brain barrier and leakage of molecules across these cells. Neurotoxicity comes from the presence of Hb, heme, and iron derived from its catabolism, as well as inflammatory events all with consequences for the different types of brain cells. In brain cells, the HO2 isozyme predominates although the heme-inducible HO1 is also present. The role for the various cell types in the response to brain injury is under active investigation. To better assess brain injury and the response to both the brain damage, markers in the CSF are being defined not just for hemolysis and heme toxicity but also for inflammation and the activation of endothelial cells. Such information will hopefully guide and improve therapeutic approaches. In this regard, iron uptake via transferrin and, intriguingly, via ferritin into the brain are influenced by gender and genotype [103].

Hemopexin is present in the CSF, likely produced by neurons and ependymal cells [22,23]. Several studies have revealed that CSF HPX levels change in response to neurological diseases including Alzheimer’s [104] as well as brain hemorrhage [24]. Once more, HPX’s role as a potential therapeutic was reinforced in a recent review on subarachnoid hemorrhage, which compared the pathophysiology in humans and rodents [105], and Hp is active in outcomes in patients after subarachnoid hemorrhage [106] or after ICH [107]. When there is impairment of the blood–brain barrier both brain and systemic HPX and Hp systems may interact. However, it is most likely that brain inflammation drives the local synthesis of HPX. Emerging treatment strategies for clearing hemoglobin/heme from the brain after intracranial bleeding were presented in a comprehensive review by Galea and colleagues [108]. Also, the clearance of heme through the inducible HO1 pathway as a treatment for ischemic stroke has recently been reviewed [109]. We refer the reader to these publications and have not presented these topics in detail here.

Further insight into the development of neurotoxicity and inflammatory conditions in the brain has now come from using the neurotoxic effects of lymphodepletion chemotherapy. Endothelial cell activation by cytokines leads to disseminated intravascular coagulation, capillary leakage, and increased blood–brain barrier permeability. When the blood–brain barrier is permeable, it no longer protects the CSF from exposure to high concentrations of systemic cytokines including IL6 and IL4 [110]. Such severe endothelial activation may lead to multiple areas of hemorrhage. Interferon-γ (IFN-γ) induces brain vascular pericytes to secrete cytokines that activates endothelial cells. Most patients developed cytokine release syndrome initiated by T cell activation with fever and hypotension that was associated with high IL-6. As described in Section 12, IL-6 can alter the response of models of liver and neuronal cells to cytoprotective heme-HPX signaling.

One of the most detailed studies on the relationship between protection of human brain cells via heme clearance by the HPX and Hp systems and activation of localized inflammatory response comes from a study by Righy and coworkers [24] on patients after either intracerebral or subarachnoid hemorrhage. Hemorrhagic stroke depletes the CSF of HPX and Hp as does subarachnoid hemorrhage as heme and iron increased in the CSF [24]. A comparison of the levels of heme, iron, HPX, and Hp in the plasma and CSF at 24, 48, and 72 h post-ictus revealed that iron levels were extremely high throughout. Plasma HPX levels were essentially maintained and Hp was lower at the 24 h time point, but the values of both varied widely including some patients who had undetectable protein levels. The CSF HPX decreased over the first 48 h post ictus, but then appeared to increase by 72 h. The Hp appeared to increase over this three-day period as it did in the plasma. Significantly, these changes were linked to survival in that plasma iron, and heme over the first 48 h were highest in the non-survivors. These data indicate that iron overload dominated perhaps more so than toxicity from heme per se. Nevertheless, extracellular Hb is clearly toxic, and when the defenses are overwhelmed, then some protection is derived from HPX. Furthermore, 3 days after admission, IL-4 levels in the CSF were higher in the survivors, whereas both IL-6 and IL-8 in the circulation were significantly increased in the non-survivors. These cytokine responses suggest that some anti-inflammatory protection was taking place in the brain. Thus, treatments for brain hemorrhage using local anti-inflammatory agents may have a protective role in addition to heme toxicity attenuation.

## 7. Hemopexin Metabolism Is Altered When Inflammation Occurs in the Brain in Non-Hemorrhagic Conditions

There is a novel compensatory immune-regulatory reflex system (CIRS) in a large population of patients diagnosed with depression and bipolar disorders who are particularly affected. Markers to demonstrate that there was activation of an immune-inflammatory response system (IRS) are being sought. In this study, there is an associated increase in pro-inflammatory M1 macrophages and T-helper (Th)-1 pro-inflammatory cytokines including IL-6 trans signaling, together with positive acute phase proteins and complement. In fact, evidence for activation of the immune system during major episodes comes from the findings of increases in T-helper type 2 cells that protect and secrete several interleukins (IL-4, -5, -9, -13, and -17). These cells are required for humoral immunity as well as regulatory T cells that act against extracellular pathogens and facilitate cell repair but can contribute to chronic disease such as allergies and asthma. A larger increase was observed in IL-4 and IL-10 production with IL-6 signaling and transcription of the sIL-1 receptor antagonist soluble IL-2, TNFα receptors, and HPX together with four acute phase reactants Hp, α-1 acid glycoprotein, α1-antitrypsin, and ceruloplasmin. (Based on clinical data, HPX is not an acute phase reactant in humans [62], although the human *HPX* gene contains an “active” IL-6 response element [111].) Therefore, this is evidence of the primary immune-inflammatory response with spontaneous recovery or in response to treatment with anti-depressants. Unfortunately, after each acute episode, the patients developed a sensitized IRS and CIRS response. Thus, there remains a need for composite biomarker(s) to estimate the relative ratio of these two responses in these and other mood disorders that clearly have a biochemical basis [112].

There is also a lack of biomarkers for chronic widespread pain. This occurs in chronic fibromyalgia syndrome that manifests as general muscle pain, tiredness, together with anxiety and depression. Even cognitive disabilities may exist. A preliminary proteome was analyzed using 2D electrophoresis of plasma samples to assess if different patterns in plasma proteins could be found that changed in response to pain or to the psychological distress. Hemopexin together with two complement components and clusterin were proteins associated with the psychological aspects of this condition. These were considered to represent immunity, iron, and lipid metabolism. On the other hand, proteins associated with metabolic and immunity process (kininogen, fibrinogen γ-chain, and ceruloplasmin) were linked to pain intensity. It seems that several psychiatric disorders have immune activation, where changes in HPX metabolism help to identify them [113]. Further study may provide new knowledge of factors that influence HPX metabolism and its levels in plasma and CSF.

## 8. Animal Models of Human Hemolytic Diseases and Conditions

Animal models of human diseases and potential therapies for them allow investigation of target tissues and cells that are not always possible in patients. As briefly summarized in the Introduction, the extracellular antioxidant protective role of HPX is only one means whereby the HPX system protects against heme toxicity. In a variety of animal models of hemolysis, the protective effect of HPX has been recently linked to increased HO1 activity in hepatocytes and other cell types. Importantly, it is the rate at which heme builds up in cells, the intracellular concentration of heme, and cofactors for HOs, which affect the extent of heme metabolism by HOs and, thus, the potential for toxicity either in response to heme or to increased ferrous iron from heme catabolism, as recently described [3]. When cells are incubated with “free” heme in vitro, even when albumin is present in culture medium, heme very rapidly accumulates in cells—within seconds and minutes. Also, because this process is unregulated compared with the endocytosis of heme-HPX, toxicity readily develops (see Section 2 above for the toxic effects of heme at ~10–25 µM or higher). Heme oxygenase enzymes are quickly induced upon entry of heme across the plasma membrane and into the nucleus. Under these conditions, we do not know what limits HO enzymatic activity or the rate of increases in ferrous iron in the cytosol or, simultaneously, carbon monoxide (CO) levels. What is a toxic threshold for intracellular heme concentrations? Does CO from heme catabolism inhibit the intracellular heme-proteins such as those in the electron transport chain as it does cytochrome P-450? Clearly, a certain amount of cytosolic ferrous iron from HOs can readily be dealt with by cells in response to the changes in the proteins of iron homeostasis via the Fe/IRP/IRE system. However, what might be a “breaking point” for toxicity? The cytosolic “labile” ferrous iron pool is ~1 µM whereas iron stored on ferritin is 0.7–0.36 mM. Certainly, defense against iron toxicity may be equally or perhaps more important than heme sequestration, for example, after brain hemorrhage in patients, ([24], see Section 6.4).

Protection of cells against heme involves both sequestration of extracellular heme as well as intracellular events that include induction of HO1 activity. Also, HO1 responds to many stimuli not heme solely. In animal and cell studies, evidence for a role for HO1 in cytoprotection often comes from the use of metal porphyrins (e.g., cobalt-, tin- or zinc-protoporphyrin IX) that have been shown to inhibit HO enzymatic activity in vitro (but also induce HO1 mRNA to very high levels). Thus, HOs have been linked to the cytoprotection of the HPX system because protective effects of HPX are “lost” in the presence of these heme analogs.

### 8.1. Hemopexin Reduces Inflamation and Descreases Oxidative Stress in Models of SCD

In mice models of SCD [114], a single infusion of human HPX (1 µmole/kg; ~14 µM) together with equimolar amounts to Hb increased HO1 in liver, skin, and kidney within 1 h. Hemopexin inhibited stasis from the Hb (less than 1% at 24 h) compared with untreated mice (10–11%). Plasma Hb and heme levels were unchanged 1 h after infusion of HPX. Also, and importantly, HPX did inhibit the induction of markers of inflammation such as inflammatory cytokines (chemokine 5), but did not affect the plasma levels of TNFα, IL-10 or IFNγ. Furthermore, HPX inhibited NFĸBphosphoP65, considered to promote stasis via activation of pro-inflammatory adhesion factors. Furthermore, the increased levels of hepatic oxidative stress indicated by the presence of hydroxynonenal were ameliorated by HPX. In previous studies from Vercellotti, Belcher, and colleagues [63], heme sequestration by HPX mobilization to the surface of endothelial cells of Weibel–Palade body protein P-selectin and von Willebrand factor that drive vaso-occlusion, a potentially lethal pathology of SCD. Because induction of hepatic HO1 and engineered hepatic over expression of HO1 inhibited stasis in these mice, the inhibitor tin-PP was administered at levels to block HO1 activity, which prevented the protection by HPX. Furthermore, CO administration to the mice before Hb fully restored protection by HPX. To support the relevance of the mice model to clinical SCD and to show directly that heme induces proteins such as P-selectin needed for vaso-occlusion, human umbilical vein endothelial cells were incubated with heme (10 µM for 30 min or histamine as a positive control), which readily induced P-selectin.

Overall, HPX was considered to inhibit stasis by inducing HO1 and, thus, raising CO levels. One challenge with mice as a model for human hemolytic conditions including SCD is that, unlike in humans, Hb is cleared via CD163 in macrophages and, thus, does not require Hp. Intriguingly, the authors point to novel hepatic uptake of Hb–Hp complexes into hepatocytes as an additional clearance mechanism that will need defining. It may be related to the process in rats documented in1972, where 85–95% of the radioactivity from ^59^Fe-Hb was present with liver parenchymal cells whether injected IV alone or with Hp [115]. The conclusion was that plasma protein replenishment therapies with both HPX and Hp might be best for SCD patients, especially those with acute chest syndrome.

Evidence is accruing for differences in the response of HPX, Hp, and HOs to intravenous Hb or Hb from intravascular hemolysis compared with Hb release after the administration of RBCs. Graw et al. [116] showed that an infusion of stored red blood cells increased hepatic HO 1 mRNA and also in kidney and spleen. In addition, HO1 levels in all these organs were increased by co-infusion with HPX (or albumin or Hp).

The excessive heme from erythrophagocytosis causes the loss of resident red-pulp macrophages. In this context, there are some unexpected relationships in mice between HO1 activity and hepatic HPX expression (that drives plasma levels), which is altered when spleen function is impaired. Spleen macrophages may die due to the toxicity of heme when HO1 is inhibited or when heme export via plasma membrane transporter FLVCR1b [117] is impaired because proteins heme such as HPX or albumin are needed as extracellular heme acceptors to drive transport down the concentration gradient. Hepatic HPX is increased significantly when HO1 is inhibited or absent as shown in studies with *HO1* knock out mice [118]. These studies showed that the status of spleen macrophages affects HPX levels both in the liver (HPX mRNA) and in the circulation. The implication of this includes whether some of the effects in mice given Hb, RBCs or in hyper-hemolytic states (after phenylhydrazine) are due to the changes in endogenous levels of HPX, especially when inhibitors of HO1 are administered. Thus, when both spleen macrophages and Kupffer cells are involved in RBC clearance, there may be changes in the regulation and activity in the various defenses against plasma Hb/heme including the HPX system compared with low levels of hemolysis (or IV heme or Hb in animal studies). In the light of observations on toxicity from globin, when HPX is present with Hb in Hp^−/−^ mice or in humans with hemolysis sufficient to have depleted Hp, there must be mechanisms in vivo that mitigate extracellular globin toxicity in Hp deficiency. Alternatively, globin precipitation is a key missing part of the complicated puzzle that is the mechanism of Hb/heme toxicity.

### 8.2. Hemopexin Protects the Blood–Brain Barrier

Using a model of cerebral ischemia-reperfusion injury in rats, focal cerebral ischemic and reperfusion, Dong and colleagues [119] showed that HPX alleviated cognitive dysfunction rapidly when injected intracerebro-ventricularly once reperfusion was initiated. This protective effect was also linked to HO1 activity because it was reversed by the HO1 inhibitor, zinc-PPIX [119]. In a similar experimental approach, HPX was protective after cerebral ischemia/reperfusion injury and protection was lost when zinc-PPIX was given [119].

Importantly, HPX in the cerebral spinal fluid (CSF) helps to maintaining the integrity of the blood–brain barrier, in part, because HPX and the induction of HO1 helps new blood vessel formation by supporting both the migration and differentiation of endothelial progenitor cells [120]. The administration of HPX allowed the rats to recover after cerebral ischemia, as assessed by the magnitude of their synaptic plasticity, but this was blocked by administration of the HO1 inhibitor ZnPPIX. Thus, HO1 activity is implicated in the protection by HPX of both the endothelial cells and blood–brain barrier integrity in rats with cerebral ischemia [120].

The HPX, LRP1/CD91 positive cells, and heme were detected from day 1 in both the clot and in the tissue surrounding the brain hematoma, and perihematoma in piglets were injected with autologous blood into the right frontal lobe of the brain [121]. The administration of the iron-chelator deferoxamine significantly decreased all of these indicators of the HPX system. Unfortunately, the full significance of protection by HPX in these studies is not yet clear because deferoxamine does not cross the blood–brain barrier. Therefore, when this chelator is reported to be effective, it is presumptive evidence for an impairment of the blood–brain barrier allowing molecules from the plasma to reach the brain and potentially vice versa.

Importantly, data from a rat model of the sports-related head injury supports that HPX is a potential diagnostic for a frequent form of traumatic brain injury—diffuse axonal injury. Biomarkers for brain damage are urgently needed because such injuries, as is now evident, occur even in recreational and professional sports. They represent a serious and complex brain injury with significant morbidity and mortality. Low linear and angular accelerations to the head [122] replicate axonal injury and hemorrhagic tears that represent the histology and neurological changes of axonal injury. Both HPX and glyceraldehyde-3-phosphate were 2 out of 58 proteins in plasma deemed potentially diagnostic for this type of brain injury.

A role in olfactory system development is perhaps one of the most intriguingly recent discoveries about HPX. Neurogenesis relies on neural stem cells of the subventricular zone that reside in a specialized niche. Abnormalities develop in the SV2/olfactory bulb pathways of HPX-null mice that impair neuroblast migration in the subventricular zone as well as the rostral migratory stream. Exogenous HPX inhibited apoptosis and promoted the migration and differentiation of cultured neural stem cells [123].

Furthermore, HPX is protective against bleeding and blood loss in trauma injury, which is the leading cause of death for the young, including infants, and in the middle aged. Trauma includes the severe injury from automobile accidents, industrial accidents, and on the battleground. These severe injuries lead to significant blood loss and internal bleeding may damage the lungs. Both blood loss and hemorrhagic shock after trauma are routinely treated with massive blood transfusions to maintain Hb oxygen levels. This may well be life saving for patients but also presents a hazard to their health because of extensive RBC lysis from blood transfusions. It is well established that RBCs deteriorate; the longer that blood and packed RBCs are stored, the more likely RBCs will lyse and release Hb leading to heme toxicity. Thus, extensive transfusions are associated with poor recovery due to the Hb/heme load for the patient and, significantly, there is an increase in organ injury. Furthermore, bacterial infection becomes more prevalent in part because nutritional immunity is overcome by the systemic heme and iron loads.

### 8.3. Hemopexin Is a Part of Nutritional Iron Defense System in the Lung

Mice have provided novel information on the dangers from heme leading to the development of pathology in the lung, which are exacerbated by bacterial infection of the lungs following, for example, trauma-induced hemorrhage [72]. Most human pathogens have multiple ways to acquire heme via surface receptors and transport systems for heme itself, or for Hb, Hb–Hp, heme-HPX and for iron-transferrin. After trauma hemorrhage, the mice were resuscitated with plasma and leukocyte-reduced RBCs that were either fresh or stored for 14 days. Two days later, their lungs were infected by administering the opportunistic pathogen, the K-strain of *Pseudomonas aeruginosa* that has many virulence genes. This Gram-negative rod bacterium is associated with bronchial infections in patients with cystic fibrosis and is predominantly responsible for the nosocomial infections in hospital intensive care units. Also, it has developed resistance to many antibiotics. As expected and linked with hemolysis, infusion of stored RBCs increased lung injury and the severity of bacterial infection determined by a higher bacterial count in the lung and increased pulmonary edema. These changes were greater than the responses to fresh RBC infusion. Changes in plasma Hb, heme, and non-transferrin bound iron were measured longitudinally over 48 h after trauma hemorrhage. When IV HPX (1 mg/kg) was administered immediately before resuscitation, the mice had significantly lower levels of Hb compared with mice exposed to stored RBCs. These data support that the increased lung injury (pulmonary edema and lung bacterial count) after resuscitation with stored RBCs is due to the heme. Following up on observations that heme can activate TLR4 driving the pathology of SCD in mice [63], it was shown that administration of HPX or pharmacological inhibition of TLR4, during the traumatic hemorrhage and resuscitation completely prevented the *P. aeruginosa*-induced mortality following resuscitation with stored RBC. The TLR4^−/−^ mice were similarly “protected”. In addition, using murine alveolar macrophage line (MS1) heme inhibited bacterial phagocytosis of macrophages via the release of the damage-associated molecular pattern molecule HMGB1, a death high mobility group box transcription factor, which is released from apoptotic cells. Antibody to HMGB1 protected against death following the *P. Aeruginosa* infection. It was shown from a group of 50 patients with trauma hemorrhage that they received sufficient heme from resuscitation therapy transfusions to overwhelm HPX. However, it remains to be established how changes over time of the different products generated during hemolysis, including heme levels, are linked to adverse outcomes in after transfusions in these types of patients.

In mice, HPX has been shown to be involved in the nutritional iron defense system in the lungs. Pneumonic plague is highly contagious and the causative agent is *Yersinia pestis.* After mice received a lethal dose of pneumonic plague, both HPX and Hp increased 48 h later in the lung and serum. However, when this lethal plague dose was given together with a single injection of EV76 live attenuated *Y. pestis,* there was a more rapid increase, within 24 h, of HPX and Hp in both lung and in serum. Some broad range antibacterial drugs are available to treat outbreaks of multi-drug resistance *Y. pestis,* but while they are effective in vitro and can be given intranasally (ideal for rapid absorption and incidentally a useful route for drug administration in humans), they are not effective in mice leading to an ~86% rate of fatality. However, a combination treatment revealed that antibiotic treatment 48 h after a previously lethal dose of *Y. pestis* treatments was extremely effective—*all the mice survived*. When the mice were checked 21 days later, there were no signs of *Y. pestis* infection in the blood or in key organs such as the spleen, liver, and lungs. These studies are exciting because they reveal that live vaccine strains can activate not only lung immunity but also systemic immunity. They also show the potential for treatment via inhalants for acute lung infections and for patients whose lungs are rapidly deteriorating as in pneumonic plague. Finally, this research shows the importance of iron and heme sequestration for nutritional immunity that is protective and demonstrates a combination therapy that is effective to treat antibiotic resistant strains of pathogenic bacteria [124].

### 8.4. Hemopexin Is Neuroprotective in Models for Intracerebral Hemorrhage

Secondary brain injury is the term used for the damage produced by extracellular heme and leads to irreversible brain damage and enduring neurological deficits. The first studies showing that HPX protects the brain were published in 2009 and used a rat ischemia-reperfusion stroke model [125]. Recently, data from mice have revealed the therapeutic potential for a clinical grade HPX in neuroprotection for intracerebral hemorrhage (ICH) [107]. Brain HPX levels were increased using a CNS-targeted recombinant adeno-associated viral vector. This organ-specific expression of HPX resulted in smaller lesions and improved functional recovery from ICH including significantly reduced hematoma volumes compared with the control rats. Furthermore, although HO1 and iron levels were not elevated, there was increased microgliosis and decreased astrogliosis and lipid peroxidation. Importantly, the elevated HPX in brain improved both central and peripheral clearance mechanisms indicating some communication and interactions between the systemic and brain clearance systems. This may represent a situation where the blood–brain barrier is “leaky”, as shown when the iron chelator deferoxamine, which does not cross the blood–brain barrier, is effective at reducing brain damage and symptoms. A role for the circumventricular organs (see Section 5) is also a possibility.

### 8.5. Hemopexin Is Induced in Response to TLR7 Activation in Models of ICH

Toll like receptors are expressed by all cells of the immune system and are integral to the innate and adaptive immune systems. Thus, TLRs are logical targets for interference by pharmacological means for development and maintenance of inflammatory conditions including inflammatory bowel disease, rheumatoid arthritis, and SLE. As described above (Section 8.3), when heme binds to toll-like receptor 4 on endothelial cells, P-selectin is induced leading to the assembly of structures on the cell surface that causes vaso-occlusion as seen in SCD [63]. The Bruton tyrosine kinase (BTK), which is needed for B-cell receptor signaling and the survival of B-cells in humans, has been identified downstream of five TLRs including TLR4 and TLR7. A BTK–LRP1 pathway has been proposed to aid in heme clearance by heme-HPX uptake into brain cells in a mouse model of ICH [126]. In this model, the stereo striatal injection of type VII collagen [69] causes traumatic brain injury that is hemorrhagic with the development of inflammation. The TLR7 activation improved neurological deficits including neurological scores for spatial learning and memory. Improvement was also seen in the physical changes to the brain such as edema, blood–brain barrier permeability and the hematoma volume. Compared with sham-operated mice, TLR7 activators increased the protein expression of HPX, the scavenger receptor LRP1/CD91 that binds heme-HPX, as well as BTK and other pathway components. Furthermore, all of these proteins were decreased in response to TLR7 inhibitors. Investigation of LRP1 as a therapeutic target in ICH is described in Section 3.3. The role of BTK in the TLR-mediated regulation of innate immunity and differences among how information from mice models and humans may affect the application to human therapeutics has recently been reviewed [126].

## 9. Divergent Evolutionary Fate of Hp and HPX Reveals the Potential for Additional Roles for HPX

The only vertebrates known to lack Hb and red blood cells are, among the notothenioids, Antarctic ice fishes. Ice fishes live in the Southern Ocean that encircles Antarctica and, to survive, they produce anti-freeze proteins, thus preventing ice crystals from forming in their thin blood. Thin blood is another adaptation because at cold temperatures, thin blood is easier to circulate. Also, ice fishes have developed large hearts and their blood vessel have large diameters [127]. A genetic accident allowed ice fish to survive when a gene needed for the assembly of Hb was completely corrupted. Although cold water offers more oxygen, fish living in cold waters do have fewer RBCs in their blood over the winter months to save energy. Sadly, now these species and the icefish are threatened by climate change and warming oceans; however, they provide a unique situation to gain insights into the function of HPX.

Compared with hundreds of red blooded notothenioids, these ice fish have very little Hp and its translation into a functional protein has actually been silenced [128]. Overtime, a degeneration of the Hp genotype manifested in a separate lineage. This was due to the distinct nonsense mutations including a deletion frame shift and a mutated poly A sequence. Significantly, the loss of Hb effectively reduced the selective constraints on Hp maintenance. Importantly, this took place without affecting either the *HPX* gene or its expression. The HPX genotype is preserved and the transcription rate of the *HPX* gene is comparable to that in the red-blooded notothenioids. The authors of this study speculate that HPX persists due to the selective pressure to maintain mitochondrial function and to capture heme released from dead and dying cells. This had been previously proposed as a function for HPX, but to what extent there exists such intracellular sources of heme-proteins from dying and apoptotic cells is unknown. In addition, these fish have little bone but do have an extensive muscular system; and, thus, HPX may be conserved because it is needed for heme reclamation in myoglobin biology.

## 10. Immune Cells as Therapeutic Targets in Intravenous Hemolysis

Evidence supports that a calcium sensor “calprotectin” on the surface of immune cells may be an important target for reducing heme-mediated inflammation in patients with IVH. Human neutrophils and monocytes constitutively express this sensor, which is a heterodimer (S100A8 and S100A9). These proteins are currently possible candidates for both diagnostics and therapeutics when inflammation is causing disease-related pathology. They participate in cytoskeletal rearrangement and arachidonic acid metabolism. When the calprotectin dimer is released from cells, they modulate the inflammatory response by stimulating leukocyte recruitment and inducing cytokine secretion. Mechanistically, S100A8 drives the NLRP3 inflammasome and leads to IL-1β secretion. Myeloid-derived alarmin and S100A8 (AKA calgranulin A) have been shown to propagate hemolytic inflammation via leukocyte priming [129]. Thus, S100A8 alone may represent a novel target to reduce inflammation in hemolytic disorders.

Human CD4+ cells were differentiated into human monocytes and used to address the question of whether heme-induced inflammation is amplified under sterile conditions. When these cells were incubated with S100A8, there was the expected increase in pro-IL1β, which required the activation of NFĸB, in turn partly dependent upon signaling via the TLR4. In addition, when these cells were incubated with heme, pro-IL1β secretion was secreted and the effect of heme was enhanced by addition of S100A8. Furthermore, S100A8 levels increased in plasma when IVH was induced in mice; and heme sequestration by HPX administration prevented this S100A8 production.

The S100A8 levels were found to be increased in human hemolytic and sickle cell anemias. With the evidence that some heme effects are amplified by autocrine S100A8 production, these studies provide a mechanism whereby leukocyte priming in the absence of bacterial infection could be harmful. Thus, S100A8 represents a potential therapeutic target to reduce heme-mediated inflammation in hemolytic disorders.

The presence of CNS trauma such as spinal cord injury (SCI) has limited therapy and the pathology of SCI is linked to the polarization of microglia to the M1 or M2 state. Heme is known to activate mouse macrophages to the pro-inflammatory M1 state [130]. Also, HPX increases at the lesion site after crush injury to the spinal cord during the time when intramedullary spinal cord hemorrhage is alleviated and is synchronously correlated with the M2 marker Arginase 1 in microglia. Loss of HPX in vivo decreased the number of “protective” M2 microglia relative to increased M1 microglia in the lesion site. This is consistent with a role for the M1 microglia in delaying recovery and exacerbating the behavioral dysfunction after SCI. In LPS-stimulated primary cultured murine microglia, the heme-binding activity of HPX led to a rapid increase in the cells in the M2 state and was associated with improvements such as less neuronal degeneration, less demyelination together with increased numbers of mature oligodendrocytes [131].

## 11. Novel Metabolite Regulation of Tissue Resident Macrophages and Bone Marrow Macrophage Populations by Heme for Iron Recycling and Body Iron Homeostasis

Senescent red blood cells are degraded daily by almost half a million macrophages and their heme iron recycled for all of the body’s iron needs (see citations within Reference [132]). This link between heme and iron metabolism is important for normal iron homeostasis but also provides a special example of how heme, derived from citric acid cycle precursors, is involved in novel metabolite regulation in pathological hemolytic diseases and conditions. For example, in hemolysis, red pulp macrophages in the spleen not only phagocytose red blood cells but likely also take up Hb–Hp complexes and heme-HPX complexes via surface receptors CD163 and LRP1/CD91, respectively. Intracellular heme levels in macrophages are therefore assumed to be normally limited by heme export by FLVCR and/or degradation by HOs. This exporter is a member of the major facilitator family of transporters and, as such, moves heme (the solute) down a concentration gradient. However, FLVCR heme export in vitro requires a heme-binding protein such as albumin or HPX to be present extracellularly to accept the heme. Presumably this is also needed in vivo *and* could be compromised in patients with hemolysis and decreased HPX or when heme-binding by circulating albumin is compromised. (Many pharmaceuticals occupy the hydrophobic site on albumin that accommodates heme.) Heme catabolism generates iron and this must be exported via ferroportin to transferrin or in macrophages via secretion on L-ferritin.

In pathological hemolysis, the increasing heme is toxic and excessive heme induces the loss of resident red-pulp macrophages. As macrophages die, the population of pulp macrophages and bone marrow cells is replenished by the relief by heme of Bach1-mediated transcriptional repression of the gene encoding the transcription factor Spi-C in monocytes [132] that drives their differentiation into macrophages. Circulating monocytes enter the spleen and upregulate *SpiC*, leading to macrophage differentiation, reestablishment of the splenic red-pulp macrophage compartment, and restoration of iron homeostasis. Thus, local signaling within tissues might be sufficient to promote the acquisition of specialized macrophage functions irrespective of their lineage. This further demonstrates a critical role of monocytes to repopulate the body with splenic red-pulp macrophages and bone marrow macrophages. Furthermore, this regulation is likely to take place at other sites of hemorrhagic damage including macrophages at atherosclerotic plaques and alveolar macrophages that regulate surfactant production. Other tissue-specific macrophages are found in bone (osteoclasts) and induction of HO1-inhibited osteoclast differentiation into osteoclasts in vitro and in vivo and may affect bone in rheumatoid patients who had increased serum bilirubin levels [133].

## 12. Evidence for Cross Talk between IL-6 and the HPX System in Models of Liver and Neuronal Cells

Evidence is mounting to show that HPX protects against heme toxicity from Hb in both sterile and infectious inflammation [26]. The cytokine IL-6 contributes to host defense via stimulation of the hepatic acute phase response, immune cell responses, and hematopoiesis. However, when IL-6 synthesis is sustained due to the perturbed regulation, chronic inflammation ensues, which is life threatening. Il-6 is elevated in several hemolytic states and in mice studies to model human sepsis [134]. Interleukin-6 interacts with specific receptors on many different cell types including hepatocytes and lymphocytes but also on bone marrow cells, synovial fibroblasts, CD4 and CD8T cells, and B cells. Interleukin-6 can activate the MAP3K upstream kinase MKK4 for the Jun N-terminal kinase (JNK) in human HepG2 hepatoma cells [135], a signaling pathway well known to regulate several normal biological processes, not solely stress-related responses. A substrate of phospho-JNK, the transcription factor c-Jun, is phosphorylated in mouse hepatoma cells in response to heme-HPX providing evidence for activation of the JNK pathway [136]. Because the HPX system is protective, we propose that JNK activation by heme-HPX is part of JNK’s important role in normal biological processes needed for the maintenance and/or restoration of cell homeostasis in hemolysis and oxidative stress.

The purpose of this research was to determine if signaling pathways activated by the inflammatory cytokine IL-6 affect, and possibly override, the activation of the JNK pathway by heme- HPX, and if so, to what extent. Our hypothesis was that if there is cross talk between the IL-6 and heme-HPX activation of the JNK pathway, then the target for inhibition by IL-6 will be MKK4 rather than JNK. Elevated IL-6 cross talk in the early stages of inflammation when there is hemolysis may reprogram the cytoprotective cell response(s) to heme-HPX and/or perhaps impair them.

**Figure 1:**
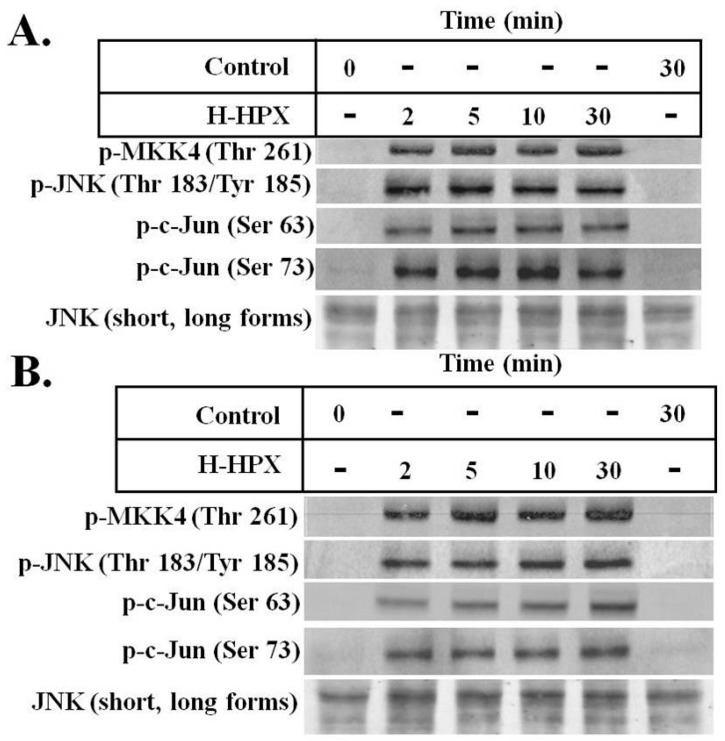
**Heme-hemopexin activates the JNK pathway via MKK4 in hepatic and neuronal cells.** In panel (**A**) Mouse Hepa cells were incubated with PBS (control) or heme-HPX (5 μM, H-HPX in PBS) for up to 30 min as indicated. The levels of p-MKK4 and p-JNK—both kinases of the JNK pathway and of p-c-Jun, a substrate of JNK, together with total JNK levels—were detected by immunoblotting of cell extracts (40 µg protein/lane), as described briefly below. (**B**) Data shown are from a similar time course experiment to determine the effects of heme-HPX on the JNK pathway and c-Jun phosphorylation in rat PC12 cells. The data shown in both panels are from a representative experiment of two independent biological replicates. Hepa cells are minimal deviation hepatoma cells from a mouse solid tumor line BW 7756 and were grown and maintained as previously described [1]. Western blots were performed by loading the same amount of protein from the sample of cell lysate per lane (40 µg protein/lane), and after electrophoresis on SDS-PAGE gels, the proteins were transferred to a nitrocellulose membrane (Bio-Rad, Hercules, CA, USA). Primary antibodies (Thermo-Fisher Scientific, Waltham, MA, USA) used at 1:1000 dilution, were detected by binding a secondary antibody of goat anti-rabbit IgG horseradish peroxidase (generally 1:5–10,000) and the peroxidase enzyme activity detected using the chemiluminescence ECL system (Amersham Pharmacia Biotech, Piscataway, NJ, USA). Data were collected using a multifunction phosphoimager/fluorimager laser scanner (Molecular Dynamic Storm Molecular Imager, LICOR, Lincoln, NE, USA).

Our preliminary data showed that incubation of heme-HPX with mouse Hepa cells as models of hepatocytes (Figure 1A) and with rat PC12 cells that are models of cholinergic neurons (Figure 1B, [137]) led to the rapid phosphorylation of MKK4 and JNK. Furthermore, two JNK substrates c-Jun(Ser63) and c-Jun(Ser73) were also phosphorylated These phosphorylations occurred very rapidly with very high levels of p-MKK4(Thr261), p-JNK(Thr183/Tyr185), and p-c-Jun(Ser63, Ser73) detectable in cell extracts within 2 min after addition of 5 µM heme-HPX to the cell medium. The time course data in Figure 1 show that they remain high for at least 30 min relative to total JNK levels supporting activation of the JNK pathway by heme-HPX.

**Figure 2:**
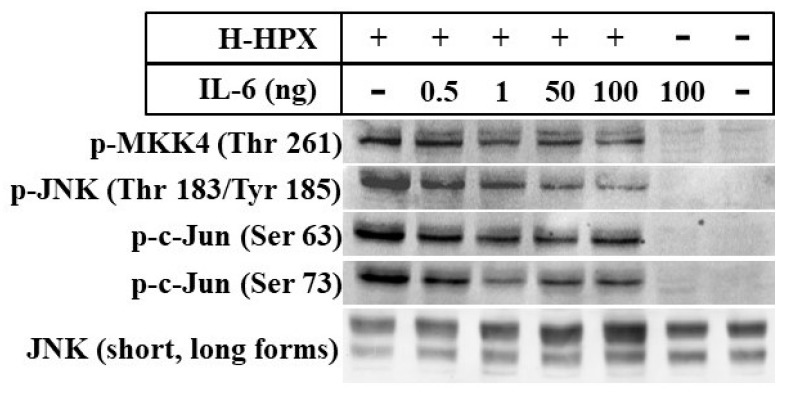
**IL-6 crosstalk decreases JNK pathway component levels induced by heme-HPX.** The data are from immunoblot analyses of cell extracts (40 µg protein/lane, as described in the legend to Figure 1). After incubation of Hepa cells with 5 µM heme-HPX in the presence or absence of IL-6 at the concentrations shown, for 30 min, IL-6 (1.0–100 ng/mL) consistently decreased to 54.3% the levels of p-MKK4 (mean +/− SD of three highest IL-6 concentration datasets: 58.63 +/− 6.21; 49.97 +/− 9.66). The IL-6 also decreased p-JNK levels to 60.4% (mean +/− SD of three highest IL-6 concentration data sets 56.25 +/− 11.56 and 64.5 +/− 4.96). These data show that IL-6 decreases the levels of key kinases of the JNK pathway activated by heme-HPX in Hepa cells. The data shown are from a representative experiment of two independent biological replicates. Kendall’s tau and Spearman’s rho rank correlation analyses were used for the statistical analyses of the relationship between IL-6 concentrations and the levels of p-MKK4 and p-JNK as described in the text.

When Hepa cells were incubated with increasing concentrations of IL-6 simultaneously with heme-HPX, the extent of phosphorylation of MKK4, JNK, and of c-Jun (Ser 63 and 73) was significantly decreased (Figure 2). The effect of IL-6 was dose-dependent. The IL-6 consistently decreased on average to 54.3% the levels of p-MKK4 and also decreased p-JNK levels to 60.4% (further details are in the legend of Figure 2). We used correlation analyses to assess for a possible association first between the IL-6 and p-MKK4 levels as variables and, second, between the IL-6 and p-JNK levels as variables to validate our hypothesis. Statistical analysis using Kendall’s Tau and Spearman’s rho rank correlation analyses show there was a strong negative correlation between the IL-6 concentration and p-MKK4 levels and also the IL-6 and p-JNK levels. Applying both types of correlation analyses the correlation was significant at the 0.01 level (2 tailed). The Kendall’s tau correlation coefficients were −0.683, significance 0.008 (2 tailed), and −0.730, significance 0.005 (2 tailed) for p-MKK4 and p-JNK, respectively, and the Spearman’s rho correlation coefficients were −0.775, significance 0.008 (2 tailed), and −0.825, significance 0.003 (2 tailed) for p-MKK4 and p-JNK, respectively. Thus, IL-6 significantly reduced JNK pathway activation by heme-HPX demonstrating for the first time cross talk on a heme-HPX signaling pathway with an inflammatory cytokine and confirming our hypothesis.

Although, MKK4 is a master regulator against liver damage and for liver regeneration [138], the implications of this initial in vitro study for the effects of IL-6 on the HPX system in vivo in clinical hemolytic and inflammatory conditions and in rodent models of HPX replenishment remain to be established. Many stimuli of hepatocytes regulate the acute phase production of IL-6, both at the autocrine and endocrine/paracrine levels [139]. For example, when the liver itself is injured, the Kupffer cells are the primary source of IL-6 that stimulates IL-6 production by parenchymal cells for the acute phase response. There may be differences in the upstream events in different cell types because IL-6 alone did not phosphorylate MKK4 in the mouse hepatoma cells as it did in human HepG2 cells. In addition, there may be differences in the response to heme-HPX in the presence of IL-6 between transformed cells and non-transformed cells. Preliminary data from PC 12 cells (not shown) revealed that IL-6 decreased pMKK4 levels in response to 5 µM heme-HPX more effectively when added to the medium 30 min before heme-HPX. This suggests some differences in response to IL-6 cross talk with heme-HPX between liver and neuronal cells.

Nevertheless, and overall, this research shows that further investigations of the interaction between IL-6 and the HPX system in different cell types including primary human hepatocytes are warranted. Our data provide a basis to gain a better understanding of the consequences of cross talk between the IL6 and other signaling pathways that may ultimately be used to improve patient diagnosis, therapy, and prognosis when there is hemolysis together with immune and inflammatory responses.

## 13. Conclusions

**Figure 3:**
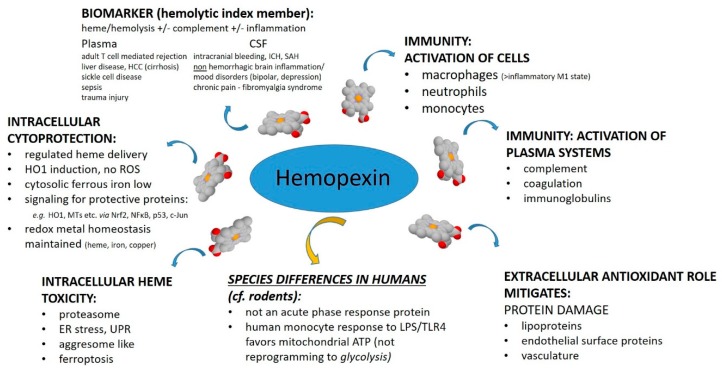
**Summary of the current status of the hemopexin (HPX) system and the role it plays in the biological processes that protect the human body against heme- and iron-mediated toxicity.** This depiction of the processes that are associated with the HPX system as it protects against heme toxicity provides a sense of the extensive nature of the biological systems and cells that are deleteriously affected by heme. Furthermore, in humans, C-reactive protein 1 and Hp levels rise when there is an acute phase reaction but HPX does not [62]. In addition, lipopolysaccharide-(LPS) stimulated human peripheral blood monocyte-derived macrophages rely on oxidative phosphorylation for ATP production and do not undergo the metabolic reprogramming to glycolysis as do mouse bone marrow-derived macrophages [140]. Such differences require assessing when using mice as pre-clinical models [141].

It is apparent from the research covered here, which builds on many previous studies in this area, that we now have a more detailed understanding of how the hemopexin system protects against both heme toxicity and iron toxicity. Many biological events are involved and include different types and sources of hemolysis; heme-driven activation of cells of the immune system and of endothelial cells; heme- and cytokine-mediators, among others, of inflammation; and specific parameters of heme and iron homeostasis, such as the activity of HOs, the induction of FtH, and the increase in plasma FtL. Many of these events and processes are depicted in Figure 3. Furthermore, identification of the activation of different kinds of cells reveals a significant number of sites in the body that can be perturbed by heme and, hence, the number of biological processes affected by hemolysis. For example, endothelial cells help to maintain the integrity of the blood–brain barrier and blood vessel walls and minimize the development of atheromas on the wall of blood vessels that can develop into hemorrhagic plaques.

Experimental evidence shows that HPX protects against all identified damage by heme in more instances than not. After endocytosis of heme-HPX by hepatocytes, HPX recycles from the liver after delivering heme [2], but because the hepatic concentration of heme alters HPX turnover, this can change plasma HPX levels [142,143]. Heme-HPX taken up into non-hepatic tissues by LRP1/CD91 a receptor that directs its ligands to lysosomes, may thus deplete HPX without generating signals for compensatory hepatic synthesis and secretion of HPX to plasma. Clearly, HPX levels decrease quite quickly after massive hemolysis (reflected by the number of heme molecules and the duration of the heme load) and the heme can take about 6 days to clear in humans before returning to normal levels [62].

Plasma exchange therapy appears poised to be a useful means to increase HPX and Hp and reduce heme toxicity. Perhaps such treatment could be a means of “respite care” for cells and organs. There is already evidence from studies on proteasomes, ER stress, and UPR as well as ALIS formation that heme-toxicity related responses can be short- or long-lived or are reversible. It will be important to establish the amount of intracellular heme from which cells can recover, how long a period of high intracellular heme and/or iron can be tolerated, and how cells return safely to homeostasis. In the future, what will be needed to counteract heme and iron toxicity are treatments that combine raising HPX (perhaps with Hp depending upon Hb levels) with certain targeted iron chelators (or other means to prevent ferroptosis) and/or a means to increase plasma FtL. We draw the reader’s attention to some challenges in the design of in vitro experiments to “mirror” heme toxicity in humans; as well as the differences between the response to heme/Hb toxicity of humans and mice (see Figure 3). It is perhaps worth remembering that experimental animals generally live out their lives in sterile conditions. Also, there are numerous differences between the response of human and mouse immune cells as pointed out in 2004 [141], and include both the innate and adaptive immune systems.

Analyses of the number and level of biomolecules in patient samples are currently extremely sensitive, thus generating a plethora of data. The plasma proteome contains thousands of proteins compared with those isolated by C18 chromatography including the normally abundant HPX [144]. Thus, future multiplexing analyses will generate so much data that very sophisticated software will be required for analysis, which may not be available to all researchers and physicians in the field. As the means to develop pathway analyses, pattern recognition networks are increasingly available through online bioinformatics programs. Ideally, a public repository for all this information is needed, which is accessible to researchers and physician–scientists to share and compare their patient data, especially for prognosis and response to treatments. In this review, juxtaposing a wide variety of studies will help researchers to more readily extend their sets of target molecules in the clinic, thus helping in the diagnosis, prognosis, and response to therapies of patients.

## References

[1] Smith A., Ledford B.E. (1988). Expression of the haemopexin-transport system in cultured mouse hepatoma cells. Links between haemopexin and iron metabolism. Biochem. J..

[2] Smith A., Morgan W.T. (1979). Haem transport to the liver by haemopexin. Receptor-mediated uptake with recycling of the protein. Biochem. J..

[3] Vanacore R., Eskew J.D., Sung L., Davis T., Smith A. (2019). Safe coordinated trafficking of heme and iron with copper maintain cell homeostasis: Modules from the hemopexin system. BioMetals Int. J. Role Met. Ions Biol. Biochem. Med..

[4] Sung L., Shibata M., Eskew J.D., Shipulina N., Morales P.J., Smith A. (2000). Cell surface events for metallothionein-1 and heme oxygenase-1 regulation by the hemopexin-heme transport system. Antioxid. Redox Signal..

[5] Vanacore R., Eskew J.D., Morales P.J., Sung L., Smith A. (2000). Role for copper in transient oxidation and nuclear translocation of MTF-1, but not of NFkB, by the hemopexin heme transport system. Antioxid. Redox Signal..

[6] Sung L., Morales P., Shibata M., Shipulina N., Smith A., Badman D.G., Bergeron R.J., Brittenham G.M. (2000). Defenses against extracellular heme-mediated oxidative damage: Use of iron and copper chelators to investigate the role of redox active iron, copper and heme in the hemopexin heme transport system. Iron Chelators: New Development Strategies.

[7] Smith A. (2000). Links between cell surface events involving redox-active copper and gene regulation in the hemopexin heme transport system. Antioxid. Redox Signal..

[8] Alam J., Smith A. (1989). Receptor-mediated transport of heme by hemopexin regulates gene expression in mammalian cells. J. Biol. Chem..

[9] Alam J., Shibahara S., Smith A. (1989). Transcriptional Activation of the Heme Oxygenase Gene by Heme and Cadmium in Mouse Hepatoma Cells. J. Biol. Chem..

[10] Chiabrando D., Marro S., Mercurio S., Giorgi C., Petrillo S., Vinchi F., Fiorito V., Fagoonee S., Camporeale A., Turco E. (2012). The mitochondrial heme exporter FLVCR1b mediates erythroid differentiation. J. Clin. Investig..

[11] Smith A., Dailey H.A. (1990). Transport of Tetrapyrroles: Mechanisms and Biological and Regulatory Consequences. Biosynthesis of Heme and Chlorophylls.

[12] Smith A., Kadish K.M., Smith K.M., Guilard R. (2011). Mechanisms of Cytoprotection by Hemopexin. Handbook of Porphyrin Science. Biochemistry of Tetrapyrroles.

[13] Smith A., Warren M.J., Smith A.G. (2006). Novel Heme-Protein Interactions: Some More Radical Than Others. Tetrapyrroles: Birth, Life and Death.

[14] Petryka Z.J., Pierach C.A., Smith A., Goertz M.N., Edwards P.S. (1977). Biliary excretion of exogenous hematin in rats. Life Sci..

[15] Crispe I.N. (2016). Hepatocytes as Immunological Agents. J. Immunol..

[16] Maude S.L., Frey N., Shaw P.A., Aplenc R., Barrett D.M., Bunin N.J., Chew A., Gonzalez V.E., Zheng Z., Lacey S.F. (2014). Chimeric antigen receptor T cells for sustained remissions in leukemia. N. Engl. J. Med..

[17] Teachey D.T., Lacey S.F., Shaw P.A., Melenhorst J.J., Maude S.L., Frey N., Pequignot E., Gonzalez V.E., Chen F., Finklestein J. (2016). Identification of Predictive Biomarkers for Cytokine Release Syndrome after Chimeric Antigen Receptor T-cell Therapy for Acute Lymphoblastic Leukemia. Cancer Discov..

[18] Davidsson P., Folkesson S., Christiansson M., Lindbjer M., Dellheden B., Blennow K., Westman-Brinkmalm A. (2002). Identification of proteins in human cerebrospinal fluid using liquid-phase isoelectric focusing as a prefractionation step followed by two-dimensional gel electrophoresis and matrix-assisted laser desorption/ionisation mass spectrometry. Rapid Commun. Mass Spectrom..

[19] Saso L., Leone M.G., Mo M.Y., Grippa E., Cheng C.Y., Silvestrini B. (1999). Differential changes in alpha2-macroglobulin and hemopexin in brain and liver in response to acute inflammation. Biochemistry.

[20] Stibler H. (1978). The normal cerebrospinal fluid proteins identified by means of thin-layer isoelectric focusing and crossed immunoelectrofocusing. J. Neurol. Sci..

[21] Morello N., Tonoli E., Logrand F., Fiorito V., Fagoonee S., Turco E., Silengo L., Vercelli A., Altruda F., Tolosano E. (2008). Hemopexin affects iron distribution and ferritin expression in mouse brain. J. Cell Mol. Med..

[22] Morris C.M., Candy J.M., Edwardson J.A., Bloxham C.A., Smith A. (1993). Evidence for the localization of haemopexin immunoreactivity in neurones in the human brain. Neurosci. Lett..

[23] Morello N., Bianchi F.T., Marmiroli P., Tonoli E., Rodriguez Menendez R., Silengo L., Cavaletti G., Vercelli A., Altruda F., Tolosano E. (2011). A role for hemopexin in oligodendrocyte differentiation and myelin formation. PLoS ONE.

[24] Righy C., Turon R., Freitas G., Japiassu A.M., Faria Neto H.C.C., Bozza M., Oliveira M.F., Bozza F.A. (2018). Hemoglobin metabolism by-products are associated with an inflammatory response in patients with hemorrhagic stroke. Rev. Bras. Terapia Intensiva.

[25] Fiorito V., Chiabrando D., Tolosano E. (2018). Mitochondrial Targeting in Neurodegeneration: A Heme Perspective. Pharmaceutical.

[26] Lin T., Sammy F., Yang H., Thundivalappil S., Hellman J., Tracey K.J., Warren H.S. (2012). Identification of Hemopexin as an Anti-Inflammatory Factor That Inhibits Synergy of Hemoglobin with HMGB1 in Sterile and Infectious Inflammation. J. Immunol..

[27] Schaer C.A., Deuel J.W., Bittermann A.G., Rubio I.G., Schoedon G., Spahn D.R., Wepf R.A., Vallelian F., Schaer D.J. (2013). Mechanisms of haptoglobin protection against hemoglobin peroxidation triggered endothelial damage. Cell Death Differ..

[28] Miller Y.I., Smith A., Morgan W.T., Shaklai N. (1996). Role of hemopexin in protection of low-density lipoprotein against hemoglobin-induced oxidation. Biochemistry.

[29] Merle N.S., Grunenwald A., Rajaratnam H., Gnemmi V., Frimat M., Figueres M.L., Knockaert S., Bouzekri S., Charue D., Noe R. (2018). Intravascular hemolysis activates complement via cell-free heme and heme-loaded microvesicles. JCI Insight.

[30] Roumenina L.T., Rayes J., Lacroix-Desmazes S., Dimitrov J.D. (2016). Heme: Modulator of Plasma Systems in Hemolytic Diseases. Trends Mol. Med..

[31] Vallelian F., Deuel J.W., Opitz L., Schaer C.A., Puglia M., Lonn M., Engelsberger W., Schauer S., Karnaukhova E., Spahn D.R. (2015). Proteasome inhibition and oxidative reactions disrupt cellular homeostasis during heme stress. Cell Death Differ..

[32] Islam M.A., Sooro M.A., Zhang P. (2018). Autophagic Regulation of p62 is Critical for Cancer Therapy. Int. J. Mol. Sci..

[33] Khandros E., Thom C.S., D’Souza J., Weiss M.J. (2012). Integrated protein quality-control pathways regulate free alpha-globin in murine beta-thalassemia. Blood.

[34] Vasconcellos L.R., Dutra F.F., Siqueira M.S., Paula-Neto H.A., Dahan J., Kiarely E., Carneiro L.A., Bozza M.T., Travassos L.H. (2016). Protein aggregation as a cellular response to oxidative stress induced by heme and iron. Proc. Natl. Acad. Sci. USA.

[35] Larsen R., Gozzelino R., Jeney V., Tokaji L., Bozza F.A., Japiassu A.M., Bonaparte D., Cavalcante M.M., Chora A., Ferreira A. (2010). A central role for free heme in the pathogenesis of severe sepsis. Sci. Transl. Med..

[36] Wang M., Kaufman R.J. (2016). Protein misfolding in the endoplasmic reticulum as a conduit to human disease. Nature.

[37] Adams C.J., Kopp M.C., Larburu N., Nowak P.R., Ali M.M.U. (2019). Structure and Molecular Mechanism of ER Stress Signaling by the Unfolded Protein Response Signal Activator IRE1. Front. Mol. Biosci..

[38] Gall T., Petho D., Nagy A., Hendrik Z., Mehes G., Potor L., Gram M., Akerstrom B., Smith A., Nagy P. (2018). Heme Induces Endoplasmic Reticulum Stress (HIER Stress) in Human Aortic Smooth Muscle Cells. Front. Physiol..

[39] Reis E.S., Mastellos D.C., Hajishengallis G., Lambris J.D. (2019). New insights into the immune functions of complement. Nat. Rev. Immunol..

[40] Merle N.S., Grunenwald A., Figueres M.L., Chauvet S., Daugan M., Knockaert S., Robe-Rybkine T., Noe R., May O., Frimat M. (2018). Characterization of Renal Injury and Inflammation in an Experimental Model of Intravascular Hemolysis. Front. Immunol..

[41] Sher E.A., Sholto A.Y., Shaklai M., Shaklai N. (2014). Can gas replace protein function? CO abrogates the oxidative toxicity of myoglobin. PLoS ONE.

[42] Bank A. (2005). Understanding globin regulation in beta-thalassemia: It’s as simple as alpha, beta, gamma, delta. J. Clin. Investig..

[43] Chen-Roetling J., Ma S.K., Cao Y., Shah A., Regan R.F. (2018). Hemopexin increases the neurotoxicity of hemoglobin when haptoglobin is absent. J. Neurochem..

[44] Chen-Roetling J., Liu W., Regan R.F. (2012). Hemopexin decreases hemin accumulation and catabolism by neural cells. Neurochem. Int..

[45] Chen-Roetling J., Regan R.F. (2016). Haptoglobin increases the vulnerability of CD163-expressing neurons to hemoglobin. J. Neurochem..

[46] Etzerodt A., Kjolby M., Nielsen M.J., Maniecki M., Svendsen P., Moestrup S.K. (2013). Plasma clearance of hemoglobin and haptoglobin in mice and effect of CD163 gene targeting disruption. Antioxid. Redox Signal..

[47] Bertaggia E., Scabia G., Dalise S., Lo Verso F., Santini F., Vitti P., Chisari C., Sandri M., Maffei M. (2014). Haptoglobin is required to prevent oxidative stress and muscle atrophy. PLoS ONE.

[48] Hirschhorn T., Stockwell B.R. (2019). The development of the concept of ferroptosis. Free Radic. Biol. Med..

[49] Dixon S.J., Lemberg K.M., Lamprecht M.R., Skouta R., Zaitsev E.M., Gleason C.E., Patel D.N., Bauer A.J., Cantley A.M., Yang W.S. (2012). Ferroptosis: An iron-dependent form of nonapoptotic cell death. Cell.

[50] Stockwell B.R., Friedmann Angeli J.P., Bayir H., Bush A.I., Conrad M., Dixon S.J., Fulda S., Gascon S., Hatzios S.K., Kagan V.E. (2017). Ferroptosis: A Regulated Cell Death Nexus Linking Metabolism, Redox Biology, and Disease. Cell.

[51] Li Q., Han X., Lan X., Gao Y., Wan J., Durham F., Cheng T., Yang J., Wang Z., Jiang C. (2017). Inhibition of neuronal ferroptosis protects hemorrhagic brain. JCI Insight.

[52] Adedoyin O., Boddu R., Traylor A., Lever J.M., Bolisetty S., George J.F., Agarwal A. (2018). Heme oxygenase-1 mitigates ferroptosis in renal proximal tubule cells. Am. J. Physiol. Ren. Physiol..

[53] NaveenKumar S.K., SharathBabu B.N., Hemshekhar M., Kemparaju K., Girish K.S., Mugesh G. (2018). The Role of Reactive Oxygen Species and Ferroptosis in Heme-Mediated Activation of Human Platelets. ACS Chem. Biol..

[54] Hirata Y., Iwasaki T., Makimura Y., Okajima S., Oh-Hashi K., Takemori H. (2019). Inhibition of double-stranded RNA-dependent protein kinase prevents oxytosis and ferroptosis in mouse hippocampal HT22 cells. Toxicology.

[55] Ayton S., Diouf I., Bush A.I. (2017). Evidence that iron accelerates ALzheimer’s pathology: A CSF biomarker study. J. Neurol. Neurosurg. Psychiatry.

[56] Lei P., Ayton S., Appukuttan A.T., Moon S., Duce J.A., Volitakis I., Cherny R., Wood S.J., Greenough M., Berger G. (2017). Lithium suppression of tau induces brain iron accumulation and neurodegeneration. Mol. Psych..

[57] Ayton S., Lei P., Hare D.J., Duce J.A., George J.L., Adlard P.A., McLean C., Rogers J.T., Cherny R.A., Finkelstein D.I. (2015). Parkinson’s disease iron deposition caused by nitric oxide-induced loss of beta-amyloid precursor protein. J. Neurosci..

[58] Zille M., Karuppagounder S.S., Chen Y., Gough P.J., Bertin J., Finger J., Milner T.A., Jonas E.A., Ratan R.R. (2017). Neuronal Death After Hemorrhagic Stroke In Vitro and In Vivo Shares Features of Ferroptosis and Necroptosis. Stroke.

[59] Imoto S., Kono M., Suzuki T., Shibuya Y., Sawamura T., Mizokoshi Y., Sawada H., Ohbuchi A., Saigo K. (2018). Haemin-induced cell death in human monocytic cells is consistent with ferroptosis. Transfus. Apher. Sci..

[60] DeGregorio-Rocasolano N., Marti-Sistac O., Gasull T. (2019). Deciphering the Iron Side of Stroke: Neurodegeneration at the Crossroads Between Iron Dyshomeostasis, Excitotoxicity, and Ferroptosis. Front. Neurosci..

[61] Smith A., McCulloh R.J. (2015). Hemopexin and haptoglobin: Allies against heme toxicity from hemoglobin not contenders. Front. Physiol..

[62] Smith A., McCulloh R.J. (2017). Mechanisms of haem toxicity in haemolysis and protection by the haem-binding protein, haemopexin. ISBT Sci. Ser..

[63] Belcher J.D., Chen C., Nguyen J., Milbauer L., Abdulla F., Alayash A.I., Smith A., Nath K.A., Hebbel R.P., Vercellotti G.M. (2014). Heme triggers TLR4 signaling leading to endothelial cell activation and vaso-occlusion in murine sickle cell disease. Blood.

[64] Vendrame F., Olops L., Saad S.T.O., Costa F.F., Fertrin K.Y. (2018). Differences in heme and hemopexin content in lipoproteins from patients with sickle cell disease. J. Clin. Lipidol..

[65] Yalamanoglu A., Deuel J.W., Hunt R.C., Baek J.H., Hassell K., Redinius K., Irwin D.C., Schaer D.J., Buehler P.W. (2018). Depletion of haptoglobin and hemopexin promote hemoglobin-mediated lipoprotein oxidation in sickle cell disease. Am. J. Physiol. Lung Cell Mol. Physiol..

[66] Nguyen T.C., Han Y.Y., Kiss J.E., Hall M.W., Hassett A.C., Jaffe R., Orr R.A., Janosky J., Carcillo J.A. (2008). Intensive plasma exchange increases a disintegrin and metalloprotease with thrombospondin motifs-13 activity and reverses organ dysfunction in children with thrombocytopenia-associated multiple organ failure. Crit. Care Med..

[67] Louie J.E., Anderson C.J., Fayaz M.F.K., Henry A., Killeen T., Mohandas N., Yazdanbakhsh K., Belcher J.D., Vercellotti G.M., Shi P.A. (2018). Case series supporting heme detoxification via therapeutic plasma exchange in acute multiorgan failure syndrome resistant to red blood cell exchange in sickle cell disease. Transfusion.

[68] Hvidberg V., Maniecki M.B., Jacobsen C., Hojrup P., Moller H.J., Moestrup S.K. (2005). Identification of the receptor scavenging hemopexin-heme complexes. Blood.

[69] Wang G., Guo Z., Tong L., Xue F., Krafft P.R., Budbazar E., Zhang J.H., Tang J. (2018). TLR7 (Toll-Like Receptor 7) Facilitates Heme Scavenging Through the BTK (Bruton Tyrosine Kinase)-CRT (Calreticulin)-LRP1 (Low-Density Lipoprotein Receptor-Related Protein-1)-Hx (Hemopexin) Pathway in Murine Intracerebral Hemorrhage. Stroke.

[70] Kushner I., Edgington T.S., Trimble C., Liem H.H., Muller-Eberhard U. (1972). Plasma hemopexin homeostasis during the acute phase response. J. Lab. Clin. Med..

[71] Drieghe S., Stove V., Decruyenaere J., Delanghe J. (2013). Interpretation of hemolysis tests following administration of a second-generation hemoglobin-based oxygen carrier. Acta Clin. Belg..

[72] Wagener B.M., Hu P.J., Oh J.Y., Evans C.A., Richter J.R., Honavar J., Brandon A.P., Creighton J., Stephens S.W., Morgan C. (2018). Role of heme in lung bacterial infection after trauma hemorrhage and stored red blood cell transfusion: A preclinical experimental study. PLoS Med..

[73] Brewin J., Tewari S., Menzel S., Kirkham F., Inusa B., Renney G., Ward M., Rees D.C. (2019). The effects of hydroxycarbamide on the plasma proteome of children with sickle cell anaemia. Br. J. Haematol.

[74] Smith A., Ferreira G. (2013). Protection Against Heme Toxicity: Hemopexin Rules, OK?. Handbook of Porphyrin Science.

[75] Handtke S., Steil L., Palankar R., Conrad J., Cauhan S., Kraus L., Ferrara M., Dhople V., Wesche J., Volker U. (2019). Role of Platelet Size Revisited-Function and Protein Composition of Large and Small Platelets. Thromb. Haemost..

[76] Yuan X.M., Ward L.J., Forssell C., Siraj N., Li W. (2018). Carotid Atheroma From Men Has Significantly Higher Levels of Inflammation and Iron Metabolism Enabled by Macrophages. Stroke.

[77] Mikuls T.R., Moreland L.W. (2001). TNF blockade in the treatment of rheumatoid arthritis: Infliximab versus etanercept. Expert Opin. Pharm..

[78] Estelius J., Lengqvist J., Ossipova E., Idborg H., Le Maitre E., Andersson M.L.A., Brundin L., Khademi M., Svenungsson E., Jakobsson P.J. (2019). Mass spectrometry-based analysis of cerebrospinal fluid from arthritis patients-immune-related candidate proteins affected by TNF blocking treatment. Arthritis Res..

[79] Ganong W.F. (2000). Circumventricular organs: Definition and role in the regulation of endocrine and autonomic function. Clin. Exp. Pharm. Physiol..

[80] Bentivoglio M., Kristensson K., Rottenberg M.E. (2018). Circumventricular Organs and Parasite Neurotropism: Neglected Gates to the Brain?. Front. Immunol..

[81] Kalapotharakos G., Murtoniemi K., Akerstrom B., Hamalainen E., Kajantie E., Raikkonen K., Villa P., Laivuori H., Hansson S.R. (2019). Plasma Heme Scavengers Alpha-1-Microglobulin and Hemopexin as Biomarkers in High-Risk Pregnancies. Front. Physiol..

[82] Su L., Pan P., Yan P., Long Y., Zhou X., Wang X., Zhou R., Wen B., Xie L., Liu D. (2019). Role of vimentin in modulating immune cell apoptosis and inflammatory responses in sepsis. Sci. Rep..

[83] Ekregbesi P., Shankar-Hari M., Bottomley C., Riley E.M., Mooney J.P. (2018). Relationship between Anaemia, Haemolysis, Inflammation and Haem Oxygenase-1 at Admission with Sepsis: A pilot study. Sci. Rep..

[84] Jung J.Y., Kwak Y.H., Kim K.S., Kwon W.Y., Suh G.J. (2015). Change of hemopexin level is associated with the severity of sepsis in endotoxemic rat model and the outcome of septic patients. J. Crit. Care.

[85] Zarjou A., Black L.M., McCullough K.R., Hull T.D., Esman S.K., Boddu R., Varambally S., Chandrashekar D.S., Feng W., Arosio P. (2019). Ferritin Light Chain Confers Protection Against Sepsis-Induced Inflammation and Organ Injury. Front. Immunol..

[86] Hershko C. (1993). Iron, infection and immune function. Proc. Nutr. Soc..

[87] Kochan I. (1973). The role of iron in bacterial infections with special consideration of host tubercle bacillus interaction. Curr. Top. Microbiol. Immunol..

[88] Wandersman C., Stojiljkovic I. (2000). Bacterial heme sources: The role of heme, hemopotein receptors and hemophores. Curr. Opin. Microbiol..

[89] Schwarz M.I., Fontenot A.P. (2004). Drug-induced diffuse alveolar hemorrhage syndromes and vasculitis. Clin. Chest Med..

[90] Aggarwal S., Ahmad I., Lam A., Carlisle M.A., Li C., Wells J.M., Raju S.V., Athar M., Rowe S.M., Dransfield M.T. (2018). Heme scavenging reduces pulmonary endoplasmic reticulum stress, fibrosis, and emphysema. JCI Insight.

[91] Aggarwal S., Lam A., Bolisetty S., Carlisle M.A., Traylor A., Agarwal A., Matalon S. (2016). Heme Attenuation Ameliorates Irritant Gas Inhalation-Induced Acute Lung Injury. Antioxid. Redox Signal..

[92] Lin J., Li J., Huang B., Liu J., Chen X., Chen X.M., Xu Y.M., Huang L.F., Wang X.Z. (2015). Exosomes: Novel biomarkers for clinical diagnosis. Sci. World J..

[93] Hourcade D.E. (2006). The role of properdin in the assembly of the alternative pathway C3 convertases of complement. J. Biol. Chem..

[94] Kodidela S., Wang Y., Patters B.J., Gong Y., Sinha N., Ranjit S., Gerth K., Haque S., Cory T., McArthur C. (2019). Proteomic Profiling of Exosomes Derived from Plasma of HIV-Infected Alcohol Drinkers and Cigarette Smokers. J. Neuroimmune Pharm..

[95] Takahashi N., Takahashi Y., Putnam F.W. (1984). Structure of human hemopexin: O-glycosyl and N-glycosyl sites and unusual clustering of tryptophan residues. Proc. Natl. Acad. Sci. USA.

[96] Ma J., Sanda M., Wei R., Zhang L., Goldman R. (2018). Quantitative analysis of core fucosylation of serum proteins in liver diseases by LC-MS-MRM. J. Proteom..

[97] Zhu J., Warner E., Parikh N.D., Lubman D.M. (2019). Glycoproteomic markers of hepatocellular carcinoma-mass spectrometry based approaches. Mass Spectrom. Rev..

[98] Elphinstone R.E., Conroy A.L., Hawkes M., Hermann L., Namasopo S., Warren H.S., John C.C., Liles W.C., Kain K.C. (2016). Alterations in Systemic Extracellular Heme and Hemopexin Are Associated With Adverse Clinical Outcomes in Ugandan Children With Severe Malaria. J. Infect. Dis.

[99] Conroy A.L., Hawkes M.T., Elphinstone R., Opoka R.O., Namasopo S., Miller C., John C.C., Kain K.C. (2018). Chitinase-3-like 1 is a biomarker of acute kidney injury and mortality in paediatric severe malaria. Malar. J..

[100] Vazquez C.M.P., Costa J.O., Bomfim L.G.S., Pires L.V., da Silva D.G., Fukutani K.F., de Jesus A.R., de Jesus Silva N., de Jesus Santana G., de Moura T.R. (2019). Oxidized Low-Density Lipoprotein (Ox-LDL) and Triggering Receptor-Expressed Myeloid Cell (TREM-1) Levels Are Associated with Cardiometabolic Risk in Nonobese, Clinically Healthy, and Young Adults. Oxid Med. Cell Longev..

[101] Hou F.Q., Lei X.F., Yao J.L., Wang Y.J., Zhang W. (2015). Tetraspanin 1 is involved in survival, proliferation and carcinogenesis of pancreatic cancer. Oncol. Rep..

[102] Lim J.H., Lee C.H., Kim K.Y., Jung H.Y., Choi J.Y., Cho J.H., Park S.H., Kim Y.L., Baek M.C., Park J.B. (2018). Novel urinary exosomal biomarkers of acute T cell-mediated rejection in kidney transplant recipients: A cross-sectional study. PLoS ONE.

[103] Wade Q.W., Chiou B., Connor J.R. (2019). Iron uptake at the blood-brain barrier is influenced by sex and genotype. Adv. Pharm..

[104] Castano E.M., Roher A.E., Esh C.L., Kokjohn T.A., Beach T. (2006). Comparative proteomics of cerebrospinal fluid in neuropathologically-confirmed Alzheimer’s disease and non-demented elderly subjects. Neurol. Res..

[105] Leclerc J.L., Garcia J.M., Diller M.A., Carpenter A.M., Kamat P.K., Hoh B.L., Dore S. (2018). A Comparison of Pathophysiology in Humans and Rodent Models of Subarachnoid Hemorrhage. Front. Mol. Neurosci..

[106] Garland P., Durnford A.J., Okemefuna A.I., Dunbar J., Nicoll J.A., Galea J., Boche D., Bulters D.O., Galea I. (2016). Heme-Hemopexin Scavenging Is Active in the Brain and Associates With Outcome After Subarachnoid Hemorrhage. Stroke.

[107] Leclerc J.L., Santiago-Moreno J., Dang A., Lampert A.S., Cruz P.E., Rosario A.M., Golde T.E., Dore S. (2018). Increased brain hemopexin levels improve outcomes after intracerebral hemorrhage. J. Cereb. Blood Flow Metab..

[108] Bulters D., Gaastra B., Zolnourian A., Alexander S., Ren D., Blackburn S.L., Borsody M., Dore S., Galea J., Iihara K. (2018). Haemoglobin scavenging in intracranial bleeding: Biology and clinical implications. Nat. Rev. Neurol..

[109] Bereczki D., Balla J., Bereczki D. (2018). Heme Oxygenase-1: Clinical Relevance in Ischemic Stroke. Curr. Pharm. Des..

[110] Gust J., Hay K.A., Hanafi L.A., Li D., Myerson D., Gonzalez-Cuyar L.F., Yeung C., Liles W.C., Wurfel M., Lopez J.A. (2017). Endothelial Activation and Blood-Brain Barrier Disruption in Neurotoxicity after Adoptive Immunotherapy with CD19 CAR-T Cells. Cancer Discov..

[111] Immenschuh S., Song D.X., Satoh H., Muller-Eberhard U. (1995). The type II hemopexin interleukin-6 response element predominates the transcriptional regulation of the hemopexin acute phase responsiveness. Biochem. Biophys. Res. Commun..

[112] Maes M., Carvalho A.F. (2018). The Compensatory Immune-Regulatory Reflex System (CIRS) in Depression and Bipolar Disorder. Mol. Neurobiol..

[113] Wahlen K., Ghafouri B., Ghafouri N., Gerdle B. (2018). Plasma Protein Pattern Correlates With Pain Intensity and Psychological Distress in Women With Chronic Widespread Pain. Front. Psychol..

[114] Belcher J.D., Chen C., Nguyen J., Abdulla F., Zhang P., Nguyen H., Nguyen P., Killeen T., Miescher S.M., Brinkman N. (2018). Haptoglobin and hemopexin inhibit vaso-occlusion and inflammation in murine sickle cell disease: Role of heme oxygenase-1 induction. PLoS ONE.

[115] Bissell D.M., Hammaker L., Schmid R. (1972). Hemoglobin and erythrocyte catabolism in rat liver: The separate roles of parenchymal and sinusoidal cells. Blood.

[116] Graw J.A., Mayeur C., Rosales I., Liu Y., Sabbisetti V.S., Riley F.E., Rechester O., Malhotra R., Warren H.S., Colvin R.B. (2016). Haptoglobin or Hemopexin Therapy Prevents Acute Adverse Effects of Resuscitation After Prolonged Storage of Red Cells. Circulation.

[117] Yang Z., Philips J.D., Doty R.T., Giraudi P., Ostrow J.D., Tiribelli C., Smith A., Abkowitz J.L. (2010). Kinetics and specificity of feline leukemia virus subgroup C receptor (FLVCR) export function and its dependence on hemopexin. J. Biol. Chem..

[118] Kovtunovych G., Eckhaus M.A., Ghosh M.C., Ollivierre-Wilson H., Rouault T.A. (2010). Dysfunction of the heme recycling system in heme oxygenase 1-deficient mice: Effects on macrophage viability and tissue iron distribution. Blood.

[119] Dong B., Yang Y., Zhang Z., Xie K., Su L., Yu Y. (2019). Hemopexin alleviates cognitive dysfunction after focal cerebral ischemia-reperfusion injury in rats. BMC Anesth..

[120] Yang Y., Dong B., Lu J., Wang G., Yu Y. (2018). Hemopexin reduces blood-brain barrier injury and protects synaptic plasticity in cerebral ischemic rats by promoting EPCs through the HO-1 pathway. Brain Res..

[121] Hu S., Hua Y., Keep R.F., Feng H., Xi G. (2019). Deferoxamine therapy reduces brain hemin accumulation after intracerebral hemorrhage in piglets. Exp. Neurol..

[122] Zhang P., Zhu S., Zhao M., Zhao P., Zhao H., Deng J., Li J. (2018). Identification of plasma biomarkers for diffuse axonal injury in rats by iTRAQ-coupled LC-MS/MS and bioinformatics analysis. Brain Res. Bull..

[123] Zhu Y., Qiu Y., Chen M., Zhang Y., Cao L., Su Z., Yuan Y., Huang A., Pu Y., He C. (2018). Hemopexin is required for adult neurogenesis in the subventricular zone/olfactory bulb pathway. Cell Death Dis..

[124] Zauberman A., Gur D., Levy Y., Aftalion M., Vagima Y., Tidhar A., Chitlaru T., Mamroud E. (2019). Post-exposure administration of a Yersinia pestis live vaccine potentiates second-line antibiotic treatment against pneumonic plague. J. Infect. Dis..

[125] Li R.C., Saleem S., Zhen G., Cao W., Zhuang H., Lee J., Smith A., Altruda F., Tolosano E., Dore S. (2009). Heme-hemopexin complex attenuates neuronal cell death and stroke damage. J. Cereb. Blood Flow Metab..

[126] Weber A.N.R., Bittner Z., Liu X., Dang T.M., Radsak M.P., Brunner C. (2017). Bruton’s Tyrosine Kinase: An Emerging Key Player in Innate Immunity. Front. Immunol..

[127] Hemmingsen E.A., Douglas E.L., Llano G.A. (1977). Respiratory and circulatory adaptations to the absence of hemoglobin in chaenichthyid fishes. Adaptations within Antarctic Ecosystems.

[128] Bilyk K.T., Zhuang X., Murphy K.R., Cheng C.C. (2019). A tale of two genes: Divergent evolutionary fate of haptoglobin and hemopexin in hemoglobinless Antarctic icefishes. J. Exp. Biol..

[129] Silveira A.A.A., Mahon O.R., Cunningham C.C., Corr E.M., Mendonca R., Saad S.T.O., Costa F.F., Dunne A., Conran N. (2019). S100A8 acts as an autocrine priming signal for heme-induced human Mvarphi pro-inflammatory responses in hemolytic inflammation. J. Leukoc. Biol..

[130] Vinchi F., Costa da Silva M., Ingoglia G., Petrillo S., Brinkman N., Zuercher A., Cerwenka A., Tolosano E., Muckenthaler M.U. (2016). Hemopexin therapy reverts heme-induced proinflammatory phenotypic switching of macrophages in a mouse model of sickle cell disease. Blood.

[131] Han D., Yu Z., Liu W., Yin D., Pu Y., Feng J., Yuan Y., Huang A., Cao L., He C. (2018). Plasma Hemopexin ameliorates murine spinal cord injury by switching microglia from the M1 state to the M2 state. Cell Death Dis..

[132] Haldar M., Kohyama M., So A.Y., Kc W., Wu X., Briseno C.G., Satpathy A.T., Kretzer N.M., Arase H., Rajasekaran N.S. (2014). Heme-mediated SPI-C induction promotes monocyte differentiation into iron-recycling macrophages. Cell.

[133] Zwerina J., Tzima S., Hayer S., Redlich K., Hoffmann O., Hanslik-Schnabel B., Smolen J.S., Kollias G., Schett G. (2005). Heme oxygenase 1 (HO-1) regulates osteoclastogenesis and bone resorption. FASEB J..

[134] Jung J.Y., Kwak Y.H., Chang I., Kwon W.Y., Suh G.J., Choi D. (2017). Protective effect of hemopexin on systemic inflammation and acute lung injury in an endotoxemia model. J. Surg. Res..

[135] Johnson G.L., Nakamura K. (2007). The c-jun kinase/stress-activated pathway: Regulation, function and role in human disease. Biochim. Biophys. Acta.

[136] Eskew J.D., Vanacore R.M., Sung L., Morales P.J., Smith A. (1999). Cellular protection mechanisms against extracellular heme: Heme-hemopexin, but not free heme, activates the N-terminal c-Jun kinase. J. Biol. Chem..

[137] Greene L.A., Aletta J.M., Rukenstein A., Green S.H. (1987). PC12 pheochromocytoma cells: Culture, nerve growth factor treatment, and experimental exploitation. Methods Enzym..

[138] Wuestefeld T., Pesic M., Rudalska R., Dauch D., Longerich T., Kang T.W., Yevsa T., Heinzmann F., Hoenicke L., Hohmeyer A. (2013). A Direct in vivo RNAi screen identifies MKK4 as a key regulator of liver regeneration. Cell.

[139] Norris C.A., He M., Kang L.I., Ding M.Q., Radder J.E., Haynes M.M., Yang Y., Paranjpe S., Bowen W.C., Orr A. (2014). Synthesis of IL-6 by hepatocytes is a normal response to common hepatic stimuli. PLoS ONE.

[140] Vijayan V., Pradhan P., Braud L., Fuchs H.R., Gueler F., Motterlini R., Foresti R., Immenschuh S. (2019). Human and murine macrophages exhibit differential metabolic responses to lipopolysaccharide—A divergent role for glycolysis. Redox Biol..

[141] Mestas J., Hughes C.C. (2004). Of mice and not men: Differences between mouse and human immunology. J. Immunol..

[142] Foidart M., Eiseman J., Engel W.K., Adornato B.T., Liem H.H., Muller-Eberhard U. (1982). Effect of heme administration on hemopexin metabolism in the rhesus monkey. J. Lab. Clin. Med..

[143] Foidart M., Liem H.H., Adornato B.T., Engel W.K., Muller-Eberhard U. (1983). Hemopexin metabolism in patients with altered serum levels. J. Lab. Clin. Med..

[144] Dufresne J., Florentinus-Mefailoski A., Bowden P., Marshall J.G. (2018). A method for the extraction of the endogenous tryptic peptides (peptidome) from human EDTA plasma. Anal. Biochem..

